# Development of a sensitive trial-ready poly(GP) CSF biomarker assay for *C9orf72*-associated frontotemporal dementia and amyotrophic lateral sclerosis

**DOI:** 10.1136/jnnp-2021-328710

**Published:** 2022-04-04

**Authors:** Katherine M Wilson, Eszter Katona, Idoia Glaria, Mireia Carcolé, Imogen J Swift, Aitana Sogorb-Esteve, Carolin Heller, Arabella Bouzigues, Amanda J Heslegrave, Ashvini Keshavan, Kathryn Knowles, Saurabh Patil, Susovan Mohapatra, Yuanjing Liu, Jaya Goyal, Raquel Sanchez-Valle, Robert Jr Laforce, Matthis Synofzik, James B Rowe, Elizabeth Finger, Rik Vandenberghe, Christopher R Butler, Alexander Gerhard, John C Van Swieten, Harro Seelaar, Barbara Borroni, Daniela Galimberti, Alexandre de Mendonça, Mario Masellis, M Carmela Tartaglia, Markus Otto, Caroline Graff, Simon Ducharme, Jonathan M Schott, Andrea Malaspina, Henrik Zetterberg, Ramakrishna Boyanapalli, Jonathan D Rohrer, Adrian M Isaacs, the Genetic FTD Initiative (GENFI), Sónia Afonso

**Affiliations:** 1 UK Dementia Research Institute at UCL, UCL Queen Square Institute of Neurology, London, UK; 2 Department of Neurodegenerative Disease, UCL Queen Square Institute of Neurology, London, UK; 3 Dementia Research Centre, Department of Neurodegenerative Disease, UCL Queen Square Institute of Neurology, London, UK; 4 Wave Life Sciences, Cambridge, Massachusetts, USA; 5 Alzheimer’s Disease and Other Cognitive Disorders Unit, Neurology Service, Hospital Clínic, Institut d’Investigacións Biomèdiques August Pi I Sunyer, University of Barcelona, Barcelona, Spain; 6 Clinique Interdisciplinaire de Mémoire, Département des Sciences Neurologiques, CHU de Québec, and Faculté de Médecine, Université Laval, Quebec City, Quebec, Canada; 7 Department of Neurodegenerative Diseases, Hertie-Institute for Clinical Brain Research and Center of Neurology, University of Tübingen, Tübingen, Germany; 8 Center for Neurodegenerative Diseases, (DZNE), Tübingen, Germany; 9 Department of Clinical Neurosciences and Cambridge University Hospitals NHS Trust and Medical Research Council Cognition and Brain Sciences Unit, University of Cambridge, Cambridge, UK; 10 Department of Clinical Neurological Sciences, University of Western Ontario, University of Western Ontario, London, Ontario, Canada; 11 Leuven Brain Institute, KU Leuven, Leuven, Belgium; 12 Laboratory for Cognitive Neurology, Department of Neurosciences, KU Leuven, Leuven, Belgium; 13 Neurology Service, University Hospitals, Leuven, Belgium; 14 Nuffield Department of Clinical Neurosciences, Medical Sciences Division, University of Oxford, Oxford, UK; 15 Department of Brain Sciences, Imperial College London, London, UK; 16 Division of Neuroscience and Experimental Psychology, Wolfson Molecular Imaging Centre, The University of Manchester, Manchester, UK; 17 Departments of Geriatric Medicine and Nuclear Medicine, University of Duisburg- Essen, University of Duisburg- Essen, Essen, Germany; 18 Department of Neurology, Erasmus Medical Centre, Rotterdam, Netherlands; 19 Neurology Unit, Department of Clinical and Experimental Sciences, University of Brescia, Brescia, Italy; 20 Fondazione IRCCS Ca' Granda, Ospedale Maggiore Policlinico, Milan, Italy; 21 Centro Dino Ferrari, University of Milan, Milan, Italy; 22 Faculty of Medicine, University of Lisbon, Lisbon, Portugal; 23 Sunnybrook Health Sciences Centre, Sunnybrook Research Institute, University of Toronto, Toronto, Ontario, Canada; 24 Tanz Centre for Research in Neurodegenerative Disease, University of Toronto, Toronto, Ontario, Canada; 25 Canadian Sports Concussion Project, Toronto, Ontario, Canada; 26 Department of Neurology, University of Ulm, Ulm, Germany; 27 Center for Alzheimer Research, Division of Neurogeriatrics, Department of Neurobiology, Care Sciences and Society, Bioclinicum, Karolinska Institutet, Solna, Sweden; 28 Unit for Hereditary Dementias, Theme Aging, Karolinska University Hospital, Solna, Sweden; 29 McConnell Brain Imaging Centre, Montreal Neurological Institute, McGill University, Montreal, Québec, Canada; 30 Department of Psychiatry, Douglas Mental Health University Institute, McGill University, Montreal, Quebec, Canada; 31 Barts and The London School of Medicine and Dentistry Blizard Institute, London, UK; 32 UCL Queen Square Motor Neuron Disease Centre, Department of Neuromuscular Diseases, UCL Queen Square Institute of Neurology, UCL, London, UK; 33 Department of Psychiatry and Neurochemistry, Sahlgrenska Academy at the University of Gothenburg, Mölndal, Sweden

**Keywords:** FRONTOTEMPORAL DEMENTIA, MOTOR NEURON DISEASE

## Abstract

**Objective:**

A GGGGCC repeat expansion in the *C9orf72* gene is the most common cause of genetic frontotemporal dementia (FTD) and amyotrophic lateral sclerosis (ALS). As potential therapies targeting the repeat expansion are now entering clinical trials, sensitive biomarker assays of target engagement are urgently required. Our objective was to develop such an assay.

**Methods:**

We used the single molecule array (Simoa) platform to develop an immunoassay for measuring poly(GP) dipeptide repeat proteins (DPRs) generated by the *C9orf72* repeat expansion in cerebrospinal fluid (CSF) of people with *C9orf72*-associated FTD/ALS.

**Results and conclusions:**

We show the assay to be highly sensitive and robust, passing extensive qualification criteria including low intraplate and interplate variability, a high precision and accuracy in measuring both calibrators and samples, dilutional parallelism, tolerance to sample and standard freeze–thaw and no haemoglobin interference. We used this assay to measure poly(GP) in CSF samples collected through the Genetic FTD Initiative (N=40 *C9orf72* and 15 controls). We found it had 100% specificity and 100% sensitivity and a large window for detecting target engagement, as the *C9orf72* CSF sample with the lowest poly(GP) signal had eightfold higher signal than controls and on average values from *C9orf72* samples were 38-fold higher than controls, which all fell below the lower limit of quantification of the assay. These data indicate that a Simoa-based poly(GP) DPR assay is suitable for use in clinical trials to determine target engagement of therapeutics aimed at reducing *C9orf72* repeat-containing transcripts.

**Key messages**

- Accurate measurement of dipeptide repeat proteins (DPRs) generated by the frontotemporal dementia and amyotrophic lateral sclerosis-causing repeat expansion in *C9orf72* will be a key tool for assessing target engagement of repeat/DPR lowering strategies in clinical trials.
- Immunoassays have been developed that can detect the poly(GP) DPR in patient cerebrospinal fluid (CSF), but as some patients’ poly(GP) levels are close to background, enhanced sensitivity may be needed.
- We report the development of an ultrasensitive CSF poly(GP) detection assay that is fit-for-purpose for clinical trials. This should allow target engagement to be assessed in the vast majority of trial participants, including those with low poly(GP) levels.

## Introduction

A GGGGCC repeat expansion in the first intron of *C9orf72* is the most common genetic cause of both amyotrophic lateral sclerosis (ALS) and frontotemporal dementia (FTD), accounting for 38% and 25% of familial cases, respectively.^1^ Healthy individuals most commonly have two repeats,^2^ while people with a *C9orf72* repeat expansion (C9FTD/ALS) can carry hundreds to thousands of repeats.^3–6^ The repeats are transcribed in both sense and antisense direction, leading to the formation of RNA aggregates termed RNA foci.^7–10^ In addition, repeat-associated non-ATG translation of the repeat expansion leads to the production of dipeptide repeat proteins (DPRs). Translation occurs in all three frames from both sense and antisense transcripts producing five different dipeptide species, poly(GA), poly(GP), poly(GR), poly(PR) and poly(PA). Therapies targeting the *C9orf72* repeat expansion such as small molecules,^11 12^ antisense oligonucleotides (ASOs),^10 13–18^ siRNAs,^19^ microRNAs^20^ and CRISPR-based approaches^21–23^ are rapidly being developed. ASOs targeting the repeat expansion or *C9orf72* transcripts have been shown to reduce both RNA foci and DPR levels in human iPSC-neurons^13 14 17^ and *C9orf72* mouse models.^10 15–18^ In order to progress therapies from the bench to the bedside, biomarkers of disease that reflect target engagement are needed. An important breakthrough was the discovery that poly(GP) can be detected in the cerebrospinal fluid (CSF) of people with C9FTD/ALS using Meso Scale Discovery (MSD) immunoassays, indicating its potential as a target engagement biomarker.^17 24^ Levels of poly(GP) in CSF were not found to correlate with clinical disease markers or neurofilament CSF levels, a non-disease specific biomarker of neurodegeneration.^17 24^ Encouragingly, ASO treatment of mouse models has been shown to lead to durable, decreased poly(GP) levels both in brain tissues and mouse CSF, and a recent study showed reduction in CSF poly(GP) levels in in a person with *C9orf72* ALS, showing that CSF poly(GP) levels could be used as a pharmacodynamic biomarker.^16–18 25^

The single molecule array (Simoa) platform measures immuno-complexes bound to microscopic beads that are isolated in arrays of microwells, large enough for a single bead. Using digital detection the Simoa platform enables single molecule detection.^26^ As poly(GP) is the most straightforward DPR to measure in CSF, we developed a sensitive, qualified poly(GP) assay using Simoa technology. Following extensive assay development and qualification we measured poly(GP) levels in CSF collected through the Genetic FTD Initiative (GENFI). In this cohort the assay had 100% sensitivity and 100% specificity and showed an eightfold difference in signal between controls and the patient with C9FTD with the lowest poly(GP) levels, indicating that it can be used as a target engagement biomarker for *C9orf72* FTD/ALS.

## Materials and methods

### GENFI participants

Fifty-five participants were recruited from GENFI, a natural history study of genetic FTD based across 27 sites in Europe and Canada.^27^ Participants included 15 symptomatic *C9orf72* expansion carriers (14 with behavioural variant FTD (bvFTD) and 1 with ALS), 25 presymptomatic *C9orf72* expansion carriers and 15 non-carrier relatives, as controls. Pathogenic *C9orf72* expansion length was defined as more than 30 repeats identified by repeat-primed PCR. Participants consisted of 23 men and 32 women, with a mean (SD) age of 49.4 (13.9) years old at sample collection. Within the disease groups: presymptomatic *C9orf72* expansion carriers, 11 men and 14 women, 41.0 (10) years old and symptomatic *C9orf72* expansion carriers, 10 men and 5 women, 64.7 (8.5) years old. Fifteen healthy controls were recruited over the same time period: 2 men and 13 women, 48.2 (11.2) years old. All people in the study underwent a clinical assessment consisting of a medical history with the participant and informant, and physical examination, with symptomatic status diagnosed by a clinician who was an expert in the FTD field.^28–32^ All participants also underwent three-dimensional T1-weighted MRI of the brain. Volumetric measures of whole brain and cortical regions were calculated using a previously described method that uses the geodesic information flow algorithm, which is based on atlas propagation and label fusion.^33^ The study procedures were approved by local ethics committees at each of the participating sites and participants provided informed written consent.

### Neurodegenerative disease controls

Twenty participants with Alzheimer’s disease (AD) were recruited from the Wolfson clinical CSF study at University College London (UCL). The cohort consisted of an approximately equal ratio of men to women, an age range of 45–80 years and an AD-like CSF biomarker profile (CSF Aβ42<630 pg/mL and CSF total tau/Aβ42≥0.88)^34^ previously quantified in clinical routine testing. Twenty participants with non-*C9orf72*-associated FTD were recruited from the Longitudinal Investigation of FTD study at UCL. Eight patients had a diagnosis of bvFTD and 12 were diagnosed with non-fluent variant primary progressive aphasia. All participants had negative genetic testing for FTD-causing mutations. The cohort consisted of 15 men and 5 women, and an age range of 53–79.

### CSF and plasma collection

CSF and plasma were collected, processed and stored in aliquots at −80°C according to standardised procedures.^35^

### NfL plasma assay

Plasma neurofilament light chain (NfL) concentration was measured in 8 matched symptomatic *C9orf72* CSF donors, 10 matched presymptomatic CSF donors and 5 matched healthy control CSF donors using the multiplex Neurology 4-Plex A kit (102153, Quanterix, Billerica, Massachusetts) on the Simoa HD-1 Analyzer following manufacturer’s instructions.

### Antibodies

Rabbit Polyclonal antibodies ‘GP57’ and ‘GP60’ were produced using a synthetic polypeptide, GP(32) as antigen and provided by Wave Life Sciences. An alternative polyclonal anti-GP antibody ‘GP6834’ was custom-made by Eurogentec, using GP(8) as antigen. The monoclonal poly(GP) antibody TALS 828.179 was obtained from the Developmental Studies Hybridoma Bank, deposited by Target ALS Foundation. Antibody details are summarised in table 1.

**Table 1:** Details of polyclonal and monoclonal antibodies tested in single molecule array poly(GP) assays. Rabbit polyclonal antibodies were affinity purified prior to biotinylation and testing

Anti-GP antibody name	Peptide used as antigen	Monoclonal/polyclonal	Source
GP57	(GP)32	Rabbit polyclonal	Custom made
GP60	(GP)32	Rabbit polyclonal	Custom made
GP6834	(GP)8	Rabbit polyclonal	Custom made
mGP	(GP)8	Mouse monoclonal	TALS 828.179

Antibody bead conjugation and biotinylation were performed as recommended by Quanterix’s Homebrew Assay Development guide. Briefly, 0.3 mL of carboxylated paramagnetic beads were conjugated with 0.2 mg/mL antibody and 0.3 mg/mL 1-Ethyl-3-(3-dimethylaminopropyl) carbodiimide with conjugation performed at 2°C–8°C. This required 80 µg of input antibody. For each biotinylation, 130 µg of antibody was used at 1 mg/mL and a 40:1 ratio of NHS‐PEG4‐biotin to antibody.

### Assay optimisation

Optimisation of the poly(GP) Simoa assay was performed by testing: two step versus three step assay design, detector antibody concentrations from 0.3 µg/mL to 1.5 µg/mL, streptavidin-β-D-galactosidase (SBG) concentrations from 50 pM to 150 pM, the inclusion of helper beads at different ratios or not at all. Multiple assay combinations were run in parallel to enable selection of optimal conditions. A GST-GP32 standard curve was prepared from two starting stocks (15 000 pg/mL and 1500 pg/mL), serially diluting down from both in diluent A (Quanterix) to create a 9-point standard curve +blank. High (140 pg/mL), middle (75 pg/mL) and low (15 pg/mL) quality control (QC) samples were prepared independently for each assay from a 1500 pg/mL stock of GST-GP32. A positive control human CSF sample from *C9orf72* expansion carriers (QC4) was created by pooling a small volume of CSF from the 40 *C9orf72* expansion carriers in the GENFI cohort.

### Curve fitting

To establish best curve fitting we followed a previously described workflow.^36^ First, heteroscedasticity (the unequal variability of a variable across a range of values of a second variable that predicts it) was assessed by plotting the SD of the average number of enzyme labels per bead (AEB) signals from the calibrators from seven assays, against their concentration (online supplemental figure S1A). As the data showed heteroscedasticity, weighting was determined by plotting log(SD of signals) against log(mean of signals) (online supplemental figure S1B). After applying linear regression and determining the slope value (k), weighting was then calculated using the following formula: Weighting=1/Y^2k^ = 1/Y^1.9474^. Curves were recalculated using four parameter logistic (4PL) and five parameter logistic (5PL), with no weighting, 1.9474, or two weighting. Curve fits were assessed using criteria that relative errors (RE) and coefficient of variation (CV) for calibrators were ±15%, and RE and CV for anchor points (1 pg/mL) were ±20%. Curve fitting with 4PL 1/Y^2^ was selected as it led to all calibrator points passing these criteria (online supplemental figure S1C).

10.1136/jnnp-2021-328710.supp1 Supplementary data

**Figure 1:**
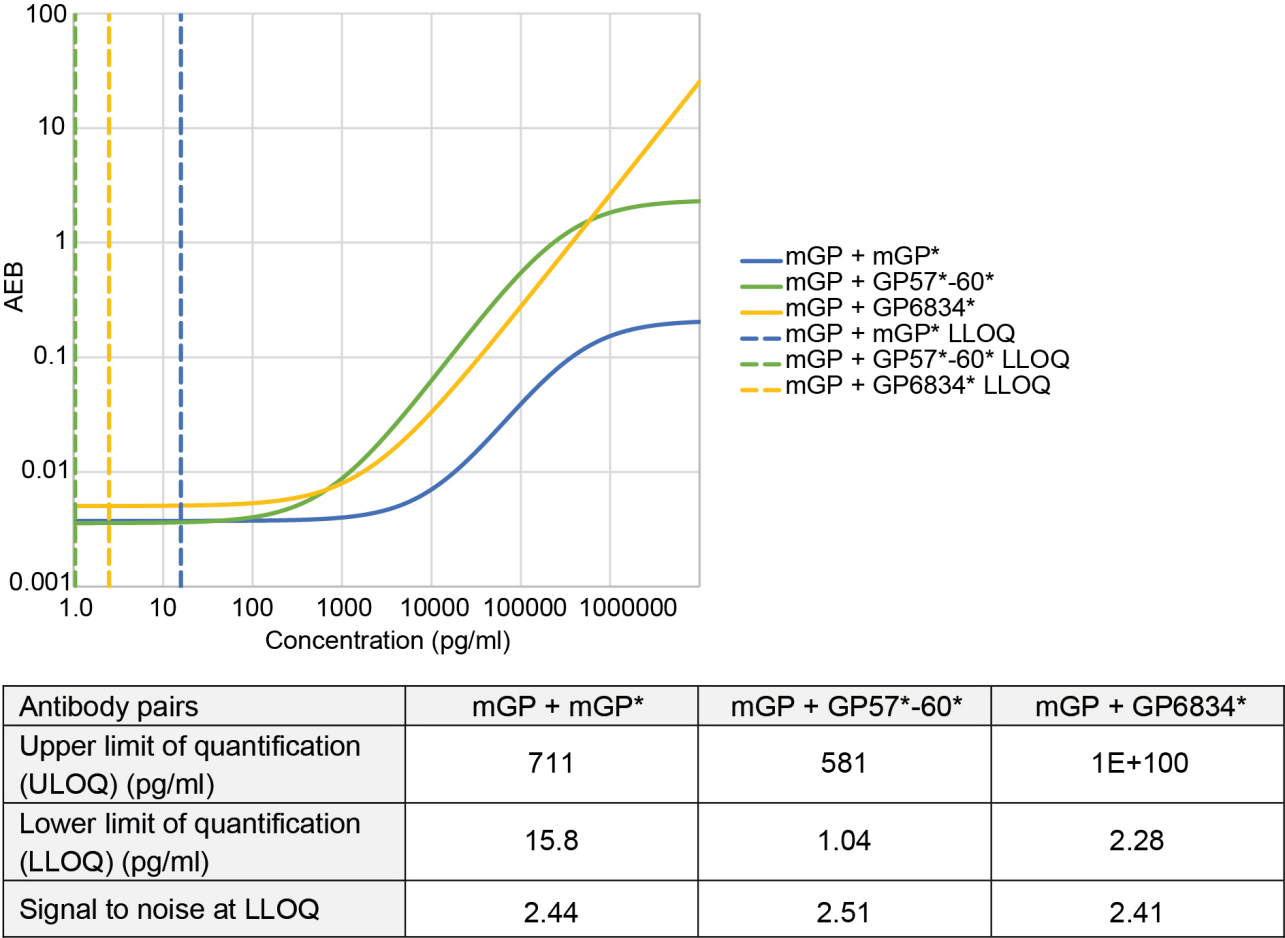
Comparison of monoclonal and polyclonal anti-poly(GP) antibodies in Simoa homebrew assays. Homebrew Simoa assay conditions were optimised using different capture antibodies and detector antibodies (*). mGP=monoclonal poly(GP) antibody (TALS 828.179). GP57*−60* is a combination of two custom polyclonal antibodies ‘GP57’ and ‘GP60’. GP6834 is an alternative custom made poly(GP) antibody. Dashed lines show predicted LLOQs for each optimised assay respectively (mGP +mGP*, mGP +GP57*−60*, mGP +GP6834*), calculated using the Quanterix assay developer tool, after running 6-point standard curves using GST-GP32 as standard. AEB, average number of enzyme labels per bead; LLOQ, lower limit of quantification; Simoa, single molecule array.

### Poly(GP) Simoa assay

The optimised Simoa assay (performed on an HD-X instrument, which is an upgraded version of the HD-1 instrument) using TALS 828.179 monoclonal antibody (mGP) beads as capture and a combination of biotinylated GP57 and GP60 (termed GP57*−60*) as detector used the following assay conditions: two-step assay, 0.3 µg/mL detector antibody (GP57*−60*), 50 pM SBG, 150 000 assay beads (mGP) with 350 000 helper beads. CSF was thawed on ice and diluted 1:2 with diluent A (Quanterix). To allow for duplicate measures 250 µL per sample was loaded into the sample plate. Analysts were blind to clinical and genetic status of samples.

Plasma samples were thawed on ice and centrifuged at 14 000 rcf for 15 min at room temperature. 125 µL was then diluted 1:1 with lysate diluent B (Quanterix) to allow duplicate measures per sample. Standard curve was prepared in lysate diluent B diluted 1:2 with control human plasma. Analysts were blind to genetic status of samples.

### Statistical analysis

Statistical analysis was carried out using GraphPad Prism software. Data were tested for normality prior to appropriate parametric or non-parametric tests. Mann-Whitney tests were used for comparing two groups, for more than two groups Kruskal-Wallis tests and Dunn’s multiple comparisons test were used. To assess correlations between poly(GP) and clinical features Spearman rho and p (two-tailed) values were calculated.

## Results

### Development of poly(GP) Simoa assay

**Figure 2:**
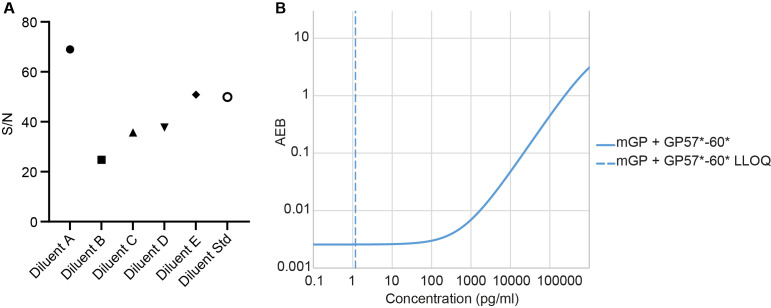
Transfer of poly(GP) assay onto Simoa HD-X. (A) Effect of sample diluents was assessed by comparing signal/noise (S/N) using control human CSF spiked with 25 pg/mL GST-GP32 standard, diluted 1 in 2 with different Quanterix diluents. Samples were run in duplicate on a single two-step Simoa assay (HD-X), using mGP +GP57*−60* Homebrew assay. (B) Standard curve produced from optimised mGP +GP57*−60* HD-X Simoa assay, using GST-GP32 as standard. LLOQ at 1.17 pg/mL shown by dashed line, calculated using the Quanterix assay developer tool. AEB, average number of enzyme labels per bead; CSF, cerebrospinal fluid; LLOQ, lower limit of quantification; Simoa, single molecule array.

To develop a sensitive poly(GP) Simoa assay we first optimised assays using the Simoa HD-1 analyser. We tested a mouse monoclonal anti-GP antibody (mGP) and a range of affinity purified rabbit polyclonal antibodies (GP57, GP60 and GP6834) raised against different length GP peptides (table 1). As the long-term goal was to have sufficient antibody quantities for use in a biomarker assay in clinical trials, we combined antibodies GP57 and GP60, which were both raised against a GP32 peptide. We found that using the monoclonal antibody as capture and the combined polyclonal antibodies as detector gave the highest signal to noise ratios for the calibrators and lowest lower limit of quantification (LLOQ) for measurement of a GST-GP32 standard peptide (figure 1). While use of mGP for both capture and detection would have been preferable, due to unlimited supply, even after assay optimisation the mGP +mGP* assay (where * indicates the biotinylated detector antibody) was over 10-fold less sensitive (LLOQ 15.8 pg/mL) than mGP +GP57*−60* (LLOQ 1.04 pg/mL) (figure 1). As mGP +GP57*−60* showed the highest sensitivity, we took this assay forward. To ensure compatibility in the long-term, we next transferred the assay to the newer Simoa HD-X platform. We found the assay required re-optimisation, with the greatest benefit gained from changing the standard curve diluent from lysate diluent B (HD-1) to diluent A (HD-X) (figure 2A). In addition, SBG was lowered from 100 pM to 50 pM for the final HD-X assay, with an LLOQ of 1.17 pg/mL (figure 2B).

### Qualification of Simoa poly(GP) assay

**Figure 3:**
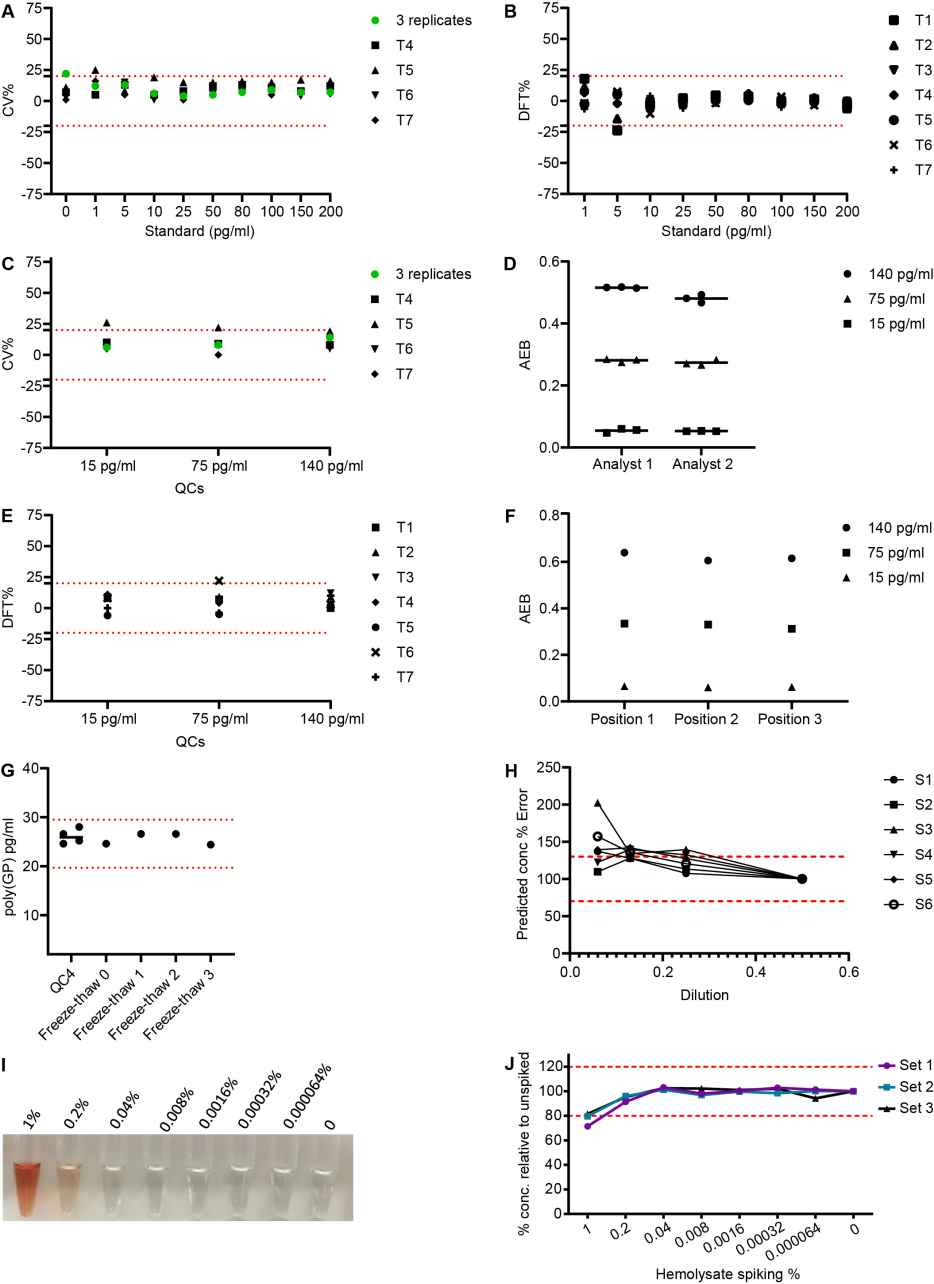
CSF poly(GP) single molecule array (Simoa) assay qualification. Ten point standard curves ranging from 200 to 1 pg/mL and three quality control (QC) samples (15 pg/mL, 75 pg/mL, 140 pg/mL) were prepared using GST-GP32 peptide and measured in seven independent assays. (A) The coefficient of variation (CV) was measured for each standard, calculating first the CV for three initial assays (green dot) and then comparing subsequent assays to the average signal from those three assays. Red dotted line at ±20% acceptance level. (B) The difference from total (DFT) calculated for each standard across seven independent assays. DFT=% difference between predicted concentration and actual concentration of calibrators. Red dotted lines at ±20% acceptance level. (C) CVs for QC samples across seven independent assays. Green dot displaying the CV from the three initial assays. Red dotted lines at ±20% acceptance level. (D) The Simoa assay signal, average number of enzyme labels per bead (AEB), measured for QCs prepared by two different analysts. Each analyst prepared three independent sets of QCs. (E) DFTs calculated for QC samples run in seven independent assays. Red dotted lines at ±20% acceptance level. (F) Intraplate variability assessed by measuring QCs in three different positions across a single assay plate. (G) Human *C9orf72* CSF donor sample (QC4) measured in four independent assays, showing high precision. Furthermore, QC4 underwent 0, 1, 2 or 3 freeze–thaw cycles prior to measurement in a single assay. Red dotted lines at ±20% acceptance level from the fresh measured QC4 sample. (H) Dilutional parallelism measured using six *C9orf72* CSF samples serially diluted, using 1 in 2 dilution as anchor. Predicted concentration % error was calculated comparing the adjusted predicted concentration at each dilution to the concentration of the 1 in 2 diluted sample (set to 100%). Red dotted lines denote ±30% from the expected predicted concentration. (I) Photo of CSF spiked with haemolysate ranging from 1% to 0.000064%. (J) CSF was spiked with haemolysate and serially diluted to give a range of equivalent % haemolysate. CSF was also spiked with 50 pg/mL GST-GP32 and poly(GP) concentration measured using the Simoa assay. Three sets were assayed and % error in predicted concentration was plotted for each sample. Red dotted lines at ±20% from expected poly(GP) concentration.

To prepare this assay for use in clinical trials it was evaluated using standard biomarker assay qualification criteria (table 2). Precision performance was assessed by analysing standard curves from seven independent assays, performed by two independent researchers. CV was <20% for all standard curve points (figure 3A and online supplemental table S1). Difference from total (DFT) (difference between predicted and actual concentration of calibrators) was below 20% for all calibrators in 6/7 assays (figure 3B and online supplemental table S2). LLOQ was identified as 1 pg/mL with upper limit of quantification at 200 pg/mL. QC samples were prepared by spiking the standard reference material GST-GP32 into diluent A. Upper QC (150 pg/mL), middle QC (75 pg/mL) and lower QC (5 pg/mL) all showed CVs <20% after seven independent runs (figure 3C and online supplemental table S3). DFTs were below 25% for QCs in seven assay runs (figure 3E and online supplemental table S4). Intraplate variability was assessed by measuring three sets of QCs across a plate within a single assay, with CV <5% for all three QCs (figure 3F and online supplemental table S5). An endogenous matrix QC sample (QC4) was generated by pooling human CSF from *C9orf72* expansion positive donors. Poly(GP) concentration of QC4 was measured in four independent assays and the CV was <20% (figure 3G and online supplemental table S7). Intermediate precision was further tested by measurement of QC samples prepared three times. This was repeated by a second analyst (figure 3D and online supplemental table S6). CV was <20% for the sets of QCs prepared independently and between the two analysts.

**Table 2:** Biomarker assay qualification criteria for poly(GP) single molecule array assay. Coefficient of variation (CV)=(SD / mean)×100. Difference from Total (DFT)=difference from predicted concentration of calibrators (pg/mL from actual, as % of actual. Quality control samples (QCs) were prepared using GST-GP32 in diluent A.

Parameter	Criteria	Achieved	Data
Precision and accuracy measuring calibrators	75% of calibrators CV≤20% and 75% of calibrators DFT≤±20%.	1×assay 89%. 6× assays 100% of calibrators CV≤20%. 1×assay 89%. 6×assays 100% of calibrators DFT ≤±20%.	Figure 3A and B. online supplemental table 1 and 2.
Precision and accuracy measuring QC samples	High (140 pg/mL), medium (75 pg/mL) and low (15 pg/mL) QCs CV ≤20% and DFT≤±20%.	6/7 assays all QCs had CV≤20%. 6/7 assays all QCs had DFT≤±20%.	Figure 3C and E. online supplemental table 3 and 4.
Intraplate and interplate reproducibility	Repeat measure of QC samples across multiple plates and positioned across a single plate CV ≤20%. Three sets QC samples prepared independently, in two independent assays by two analysts, CV ≤20% and DFT≤±20%.	100% of repeat measures of QC samples CV ≤20%. 100% of QC sets, prepared by two analysts CV≤20% and DFT≤±20%.	Figure 3D and F. online supplemental table 5 and 6.
Precision measuring matrix control sample	Repeated measures of a positive human *C9orf72* CSF sample should have CV≤20%.	Raw AEB and predicted GP concentration from four assays CV≤20%.	Figure 3G. online supplemental table 7.
Dilutional parallelism	At least three of diluted samples within the assay’s range should have DFT within ±30.0%	Using 1:2 as anchor, 4/6 samples at 1:4 had DFT within ±30.0%	Figure 3H. online supplemental table 8 and figure 1.
Freeze–thaw stability	Freeze–thaw stability of matrix control QC. CV ≤25% and DFT ≤±30%. Freeze–thaw stability of calibrators CV≤20%.	After three Freeze–thaw cycles matrix control QC CV≤25% and DFT≤±30%. After three freeze–thaw cycles of calibrators 100% CV ≤20%.	Figure 3. online supplemental table 8 and 10.
Haemoglobin tolerance	Assay should tolerate low levels of haemoglobin within ±20%.	Assay tolerates 0.2% haemolysate spike with measures within ±20%.	Figure 3I and J. online supplemental figure 2.

AEB, average number of enzyme labels per bead.

Dilutional parallelism was assessed by running CSF from six *C9orf72* expansion positive donors either neat, 1:2, 1:4, 1:8 and 1:16 in diluent A. Poly(GP) was detected above background for all dilutions. Using 1:2 as an anchor point the average % error of 4 out of 6 samples had <30% error at 1:4 dilution, passing qualification criteria (figure 3H). The percentage error increased above 30% for the majority of samples at 1:8 and 1:16 (online supplemental table S8 and figure S2). We chose to run samples at 1:2 dilution and recommend further assessment of parallelism within trials with more samples. Freeze–thaw stability of poly(GP) in CSF was tested using QC4 and measuring poly(GP) after 1, 2, and 3 freeze–thaw cycles. The signal and concentration measured had CVs of 4% and 5% respectively indicating no effect of freeze–thaw on detection of endogenous poly(GP) (figure 3G and online supplemental table S9). The freeze–thaw stability of the standard (GST-GP32) was also assessed after 1, 2, or 3 freeze–thaw cycles. Eight of the calibrators passed criteria with CV <20% and DFT <20% (online supplemental table S10). The lowest standard curve point, 1 pg/mL gave a higher DFT after three freeze–thaw cycles, but this is explained by the higher CV in signal measured for the blank in this set of calibrators, and we therefore concluded that it is unlikely that up to three freeze–thaw cycles affects the signal from GST-GP32.

During CSF collection it is possible for blood to contaminate the collected CSF. We tested if haemoglobin interfered with poly(GP) detection. We spiked a range of haemolysate concentrations (figure 3I) into control CSF and spiked with either 5 pg/mL or 50 pg/mL GST-GP32. 5 pg/mL GST-GP32 spiked in CSF was not affected by any of the haemolysate concentrations tested (online supplemental figure S3). The measurement of 50 pg/mL GST-GP32 spiked in CSF was inhibited (>20%) by addition of 1% haemolysate (figure 3J). At this concentration of haemoglobin, the CSF is visibly red (figure 3I), so samples can be excluded from analysis by appearance if required. Note, none of the CSF samples measured in this study had a red or pink appearance.

### Measurement of poly(GP) in CSF from *C9orf72* expansion carriers using the optimised, qualified Simoa assay

**Figure 4:**
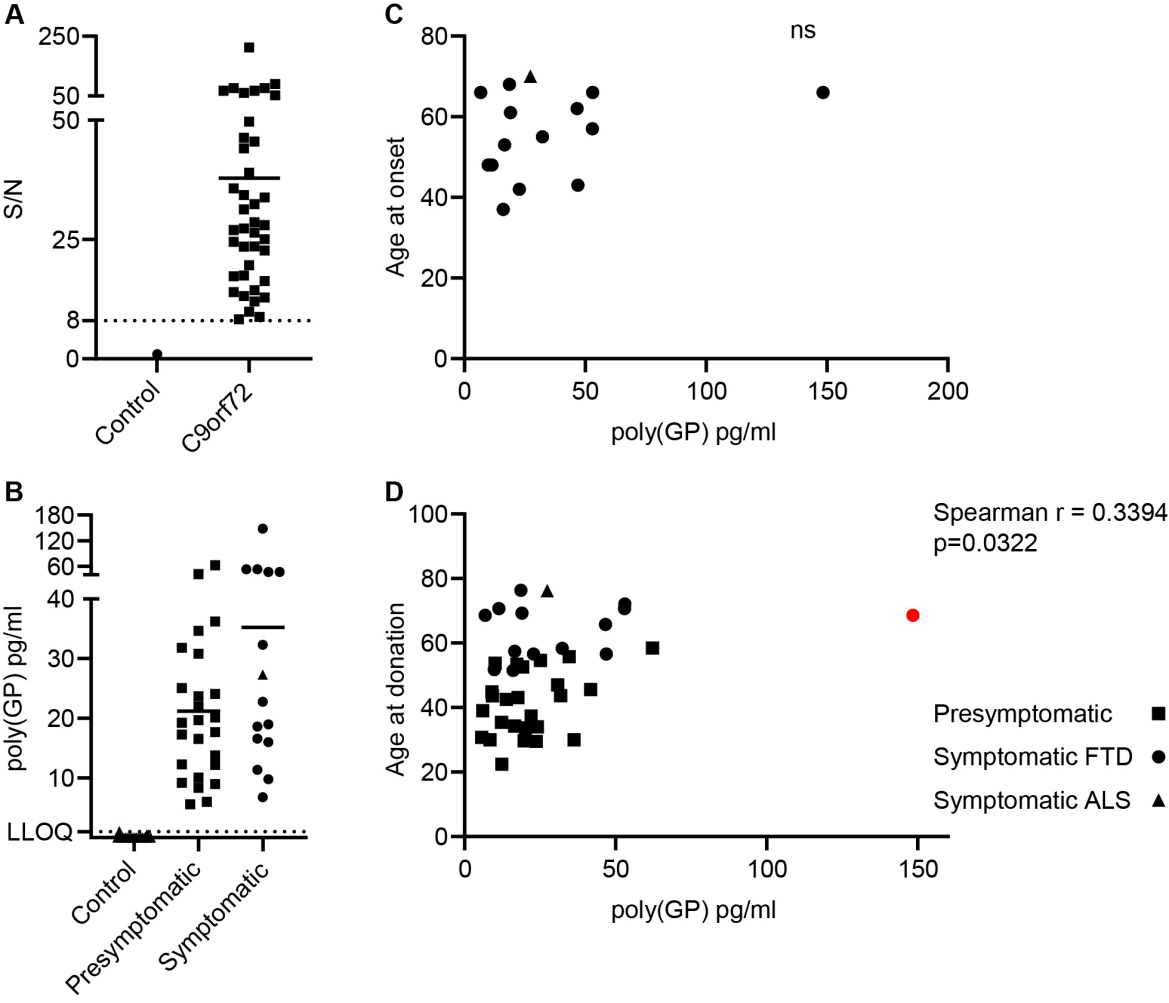
Poly(GP) levels in CSF from C9orf72 expansion carriers. Poly(GP) levels in CSF from 25 presymptomatic *C9orf72* expansion carriers, 15 symptomatic *C9orf72* carriers and 15 healthy aged matched controls were measured using our optimised Simoa HD-X assay. (A) Signal/noise (S/N) was calculated by dividing the mean AEB signal from duplicate measures of 40 *C9orf72* expansion carriers, by the mean AEB signal of CSF from all 15 healthy controls (plotted here as 1). *C9orf72* expansion carriers had poly(GP) assay signals distinct from healthy controls, with all S/N values above 8. (B) Comparison of poly(GP) levels in presymptomatic and symptomatic *C9orf72* expansion carriers. Fourteen bvFTD cases shown as circles and one ALS case shown as a triangle. Each data point is the average from a duplicate measure from each donor, with bar at mean for each group. Lower limit of quantification (LLOQ) at 1 pg/mL is shown with dotted line, determined by the lowest calibrator tested with acceptable % CV in the assay run. There is no statistical difference in poly(GP) levels between presymptomatic and symptomatic *C9orf72* expansion carriers (Mann-Whitney U test). (C) Age of onset plotted against poly(GP) pg/ml in CSF for 15 symptomatic *C9orf72* expansion carriers. Fourteen bvFTD cases shown as circles and one ALS case shown as a triangle. ns=not significant, no correlation found (Spearman r). (D) Age at donation plotted against CSF poly(GP) levels. Fourteen bvFTD cases shown as circles, one ALS case shown as a triangle and 25 presymptomatic cases shown as squares. Red dot indicates high poly(GP) CSF case, which if removed increases p value to p=0.0522. ALS, amyotrophic lateral sclerosis; AEB, average number of enzyme labels per bead; bvFTD, behavioural variant FTD; CSF, cerebrospinal fluid; CV, coefficient of variation; FTD, frontotemporal dementia; Simoa, single molecule array,

We used this sensitive, qualified assay to measure poly(GP) in a cohort of CSF from healthy controls (N=15) and *C9orf72* expansion positive donors (N=40) (demographic details in online supplemental table S11). The assay signal from the lowest *C9orf72* case had signal/noise eightfold over the average signal from control samples, showing a clear separation from signals of control CSF (figure 4A). On average the signal to noise of *C9orf72* cases versus controls was 38-fold. Poly(GP) in CSF from healthy donors was below detection level for 13 out of 15 samples or below LLOQ of the assay for the remaining 2 out of 15 cases. As poly(GP) was detected above LLOQ in all *C9orf72* cases and in no healthy controls, sensitivity and specificity were both 100%. Poly(GP) measures ranged from 6 to 148 pg/mL in *C9orf72* expansion positive donors. Despite the increased sensitivity of this Simoa assay, the levels of poly(GP) were not statistically different between presymptomatic and symptomatic *C9orf72* expansion positive donors (symptomatic mean=35.2 pg/mL, presymptomatic mean=21.2 pg/mL, p=0.1348 Mann-Whitney test), although we observed the same trend observed by others towards higher levels in symptomatic cases^17 24 37^ (figure 4B). We found no difference in poly(GP) levels between male and female *C9orf72* expansion positive donors (online supplemental figure S4A). We found no correlation between CSF poly(GP) levels and age of onset of symptomatic *C9orf72* expansion positive donors (n=15) (figure 4C). Interestingly there was a significant, moderate positive correlation (r=0.3643) between age at donation and poly(GP) measured in CSF, analysing all 40 *C9orf72* expansion positive cases (figure 4D). However, if the case with the highest poly(GP) level is removed from analysis the p value changes to p=0.0522.

Further disease control CSF samples (Alzheimer’s disease, n=20; non-*C9orf72* FTD, n=20) (online supplemental table S11) were measured using the Simoa poly(GP) assay (online supplemental figure S5). Fresh antibody-coupled beads were prepared for these additional samples and a set of standard calibrators were included to test the performance of the assay run. As expected, all samples were below the LLOQ (online supplemental figure S5).

Where data were available we also tested for correlations between CSF poly(GP) levels and both total brain and lobar volumes. No correlation was found, analysing all *C9orf72* expansion carriers or selecting symptomatic cases only (online supplemental figure S6), consistent with a previous report.^37^ Plasma NfL is a known biomarker of neurodegeneration. Plasma levels of NfL were measured in 18 of the *C9orf72* expansion carrier CSF donors (including eight symptomatic donors). As expected, plasma NfL levels were significantly higher in symptomatic carriers (online supplemental figure S7A). No correlation was found between CSF poly(GP) and plasma NfL levels analysing the small sample of eight symptomatic cases (online supplemental figure S7B).

We next optimised our poly(GP) Simoa assay for analysis of plasma. Despite the high sensitivity of the Simoa platform we were unable to detect poly(GP) in plasma from *C9orf72* expansion positive donors. Signals were below LLOQ and there was no difference between control-positive and *C9orf72*-positive signals (online supplemental figure S7C). The two cases of plasma from *C9orf72* expansion carriers which had higher AEB signals were not the same donors with higher than average CSF poly(GP), and there was no correlation between plasma AEB signal and poly(GP) measured in matched CSF samples (online supplemental figure S7D). There is a predicted 200-fold drop in concentration of NfL measured between CSF and plasma. The levels of poly(GP) in CSF were on average 26 pg/mL, so if a similar reduction is observed for poly(GP) a platform capable of detecting in femtogram range maybe required to measure poly(GP) in plasma.

## Discussion

We describe the development and qualification of a sensitive Simoa assay for poly(GP) DPRs in CSF. Multiple antibodies were assessed and compared in combinations in a Homebrew Simoa assay, identifying differences in performance across antibody combinations. In our experience not all polyclonal antibodies behave the same, even when the same peptide sequence was used for antigen. We tested the performance of a monoclonal antibody as both capture and detector in a Homebrew Simoa assay. Unfortunately, the monoclonal antibody tested here did not perform as well as a detector antibody as the polyclonal antibodies, with much higher predicted LLOQs. The reason for this difference is unclear, but the different polyclonal antibodies may recognise different secondary structures of poly(GP).

We used our qualified poly(GP) assay to analyse CSF from a small cohort of CSF samples provided by GENFI, including 15 healthy controls and 40 *C9orf72* expansion carriers. Similar to previously published studies,^17 24 37^ our assay was able to distinguish controls and *C9orf72* expansion carriers. In this cohort we had 100% sensitivity and 100% specificity with poly(GP) measured in CSF from all *C9orf72* expansion carriers, while controls either measured below detection (13/15) or below limit of quantification (2/15), determined at 1 pg/mL. *C9orf72* expansion carriers had a range of poly(GP) from 6 to 148 pg/mL, with all positive sample signals at least eightfold higher than control signals, showing a clear separation of controls from *C9orf72* expansion samples. We did not detect poly(GP) above LLOQ in Alzheimer’s disease or patients with non-*C9orf72* FTD. All previous studies used MSD immunoassays and reported the average CSF polyGP signal to be in the low nanogram range,^17 37^ while our assay gives average polyGP levels in the low–medium picogram range. This difference may be attributed to the different calibrators used in the studies, as we have noted that the same antibody can report different concentrations depending on the calibrator used. The use of different calibrators precludes a direct comparison of the different assays. Simoa technology allows detection of single molecules by converting signal from individual beads into a digital output, which we predict will provide higher sensitivity than the MSD assays that rely on an analogue output from each sample well. Although Simoa assays will not be more sensitive than MSD assays in all cases, as this will depend on the specific antibodies used, we do observe higher sensitivity compared with our standard polyGP MSD assay.^11 38 39^ A limitation of our study is that we did not carry out robustness analysis, defined as the capacity of the assay to withstand small but deliberate changes in method parameters such as incubation times, temperatures and buffer pH.^40^

In our cohort of samples we found, similar to previous studies,^17 24^ that compared with presymptomatic carriers, symptomatic carriers had higher levels of poly(GP) comparing mean levels, but this difference was not significant. As we observed a trend towards higher polyGP levels with increasing age at donation, the older age of symptomatic carriers may contribute to this effect, although we note that polyGP levels were shown to remain stable on longitudinal testing over 18–24 months.^17^ Meeter *et al* 37 found levels in symptomatic carriers were significantly higher.^37^ This may be due to the larger cohort size tested with more symptomatic donors with higher than average poly(GP) levels included. Within our small cohort there was one symptomatic *C9orf72* carrier with much higher poly(GP) levels than the rest. Age at onset (66 years) and age at donation (68 years) were both within 1 SD from the mean of other symptomatic donors, indicating no effect of higher levels of poly(GP) on these parameters. We did not have repeat length data for this cohort, although given the variability in repeat length between different tissues in the body it would be difficult to interpret repeat length data determined from blood DNA. Lehmer *et al* found no correlation between repeat size and CSF poly(GP) levels in 11 cases where DNA was available.^24^ Should postmortem tissue become available from donors in this cohort, it would be interesting to determine repeat length from brain tissue as well as measure propensity of DPR aggregates in the brain to see if poly(GP) CSF levels reflected aggregate burden.

Similar to previous studies we found no correlations between CSF poly(GP) levels and clinical features including; gender, age of onset or brain volume, analysing either total *C9orf72* cases or just symptomatic *C9orf72* carriers.^17 24 37^ We did observe a correlation between CSF poly(GP) levels and age at donation, which is potentially consistent with a relationship between *C9orf72* expansion length and age at DNA sample collection.^41^ We analysed NfL levels in a subset of donor matched plasma samples. As expected, symptomatic carriers had higher NfL plasma levels than presymptomatic or controls. As in previous studies that measured NfL in CSF,^24 37^ NfL plasma levels did not correlate with poly(GP) CSF levels. Despite the ability of the Simoa assays to detect at single-molecule levels, we were unable to measure poly(GP) in donor matched plasma samples. Signals for all samples were below quantification and did not correlate with poly(GP) CSF levels. If poly(GP) produced in the brain is present in plasma it will require a more sensitive assay platform and a better understanding of potential matrix effects. In summary, we show utility of the Simoa HD-X platform for detecting poly(GP) in the CSF of people with a *C9orf72* expansion, with assay reliability good enough to be used for target engagement analysis in clinical trials directly targeting *C9orf72* repeat containing transcripts.

## Data Availability

Data are available upon reasonable request.
