# Supplementary material for: Development of a sensitive trial-ready poly(GP) CSF biomarker assay for C9orf72-associated frontotemporal dementia and amyotrophic lateral sclerosis
Source: J Neurol Neurosurg Psychiatry. 2022 Apr 4;93(7):761–71. doi: 10.1136/jnnp-2021-328710 (PMC9279742; doi:10.1136/jnnp-2021-328710)
Supplement: Supplementary data [file jnnp-2021-328710supp001.pdf]

Development of a sensitive trial-ready poly(GP) CSF biomarker assay for *C9orf72*-associated frontotemporal dementia and amyotrophic lateral sclerosis

Katherine M Wilson[1,2*], Eszter Katona [1,2*], Idoia Glaria[1,2], Mireia Carcolé[1,2], Imogen J. Swift[1,3], Aitana Sogorb-Esteve[1,3], Carolin Heller[1,3], Arabella Bouzigues[1], Amanda J Heslegrave[1], Ashvini Keshavan[3], Kathryn Knowles[1,3], Saurabh Patil[4], Susovan Mohapatra[4], Yuanjing Liu[4], Jaya Goyal[4], Raquel Sanchez-Valle[5], Robert Laforce[6], Matthis Synofzik[7,8], James B. Rowe[9], Elizabeth Finger[10], Rik Vandenberghe[11,12,13], Christopher R. Butler[14,15], Alexander Gerhard[16,17], John C. van Swieten[18], Harro Seelaar[18], Barbara Borroni[19], Daniela Galimberti[20,21], Alexandre de Mendonça[22], Mario Masellis[23], M. Carmela Tartaglia[24], Markus Otto[25], Caroline Graff[26,27], Simon Ducharme[28,29], Jonathan M Schott[3], Andrea Malaspina[30,31], Henrik Zetterberg[1,32], Ramakrishna Boyanapalli[4‡], Jonathan D Rohrer[1,3‡], Adrian M Isaacs[1,2‡], on behalf of the Genetic FTD Initiative (GENFI)[a]

Supplemental Information

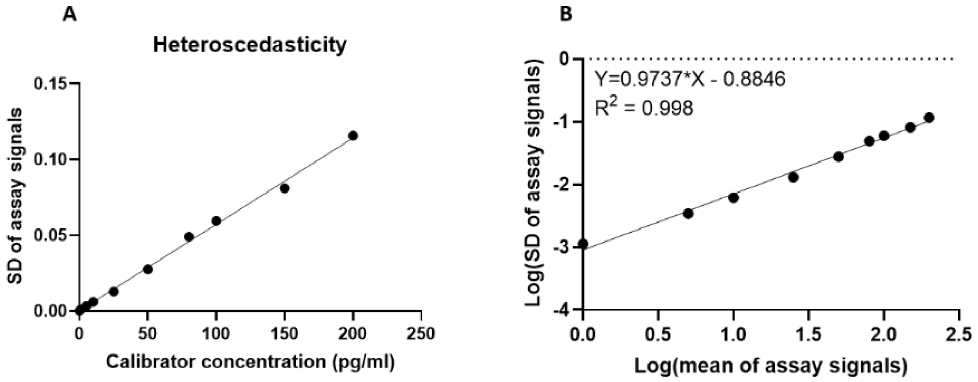

C

4PL- 1/y^2		5PL- 1/y^2		4PL- 1/y^1.9474		5PL- 1/y^1.9474		4PL- 1/y^none		5PL- 1/y^none	
%RE	CV%	%RE	CV%	%RE	CV%	%RE	CV%	%RE	CV%	%RE	CV%
**-2.21**	**2.2**	-2.21	2.1	-0.50	0.8	-0.79	1.0	-0.50	0.7	-0.71	1.1
**0.19**	**2.1**	0.19	2.1	1.14	2.0	1.81	2.3	13.62	39.4	1.81	2.3
**-0.59**	**2.5**	-0.59	2.5	-0.71	2.7	0.76	2.5	-0.71	2.8	0.56	2.6
**3.32**	**2.1**	3.34	2.1	2.59	1.9	3.86	2.6	2.50	1.9	3.79	2.7
**1.37**	**2.3**	1.49	2.4	-0.77	2.7	0.17	4.9	-1.03	2.3	0.20	4.9
**-1.37**	**2.3**	4.40	16.1	-5.43	5.5	-5.49	7.9	-6.00	4.8	-5.20	7.7
**-2.89**	**4.5**	-2.81	4.6	-7.09	9.4	-7.93	12.0	-7.86	9.1	-7.36	11.7
**-2.89**	**11.4**	-2.89	11.4	-0.20	7.7	0.89	5.1	-0.74	7.9	0.86	5.0
**2.93**	**9.3**	2.81	9.1	*38.15*	*29.1*	*50.25*	*93.9*	*37.07*	*35.2*	*50.25*	*93.9*

Supplementary figure 1. Assessment of curve fitting. A) Assessment of heteroscedasticity of data carried out by plotting standard deviation of assay signals (AEB) from the calibrator curve standards from 7 independent assays, against the calibrator concentration (pg/ml). B) To calculate weighting, linear regression was applied after plotting Log(standard deviation of assay signals) against Log(mean of assay signals) and the slope of the line (k) used in the formula: weighting= $1/Y^{2k}$. C) Curves were recalculated using 4PL and 5PL, with no weighting, 1.9474, or 2 weighting. Curve fits were assessed using criteria that % cumulative relative errors (RE%) and CV% for calibrators were +/- 15%, and RE% and CV% for anchor points (1 pg/ml) were +/- 20%. When 4PL $1/Y^2$ was used for curve fitting, all calibrator points passed these criteria and 4PL $1/Y^2$ was therefore chosen.

Wilson KM, *et al. J Neurol Neurosurg Psychiatry* 2022; 93:761–771. doi: 10.1136/jnnp-2021-328710

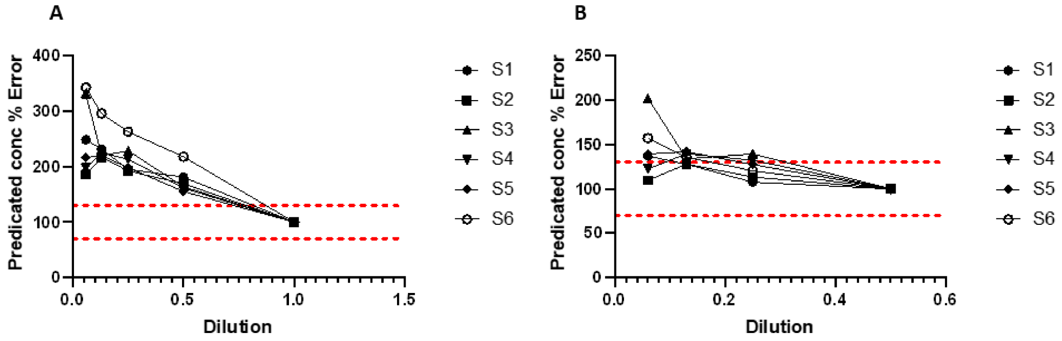

Supplementary figure 2. Dilutional parallelism. CSF from six *C9orf72* expansion positive donors was measured either neat, 1:2, 1:4, 1:8 and 1:16 diluted in diluent A. The mean AEB from duplicate measures was used to predict concentration at each dilution. **A**) The neat sample concentration was used as anchor and the % error was calculated comparing the adjusted predicted concentration at each dilution to the concentration of the neat sample. **B**) The 1:2 diluted sample used as anchor instead. Red dotted lines denote +/- 30% from the expected predicted concentration.

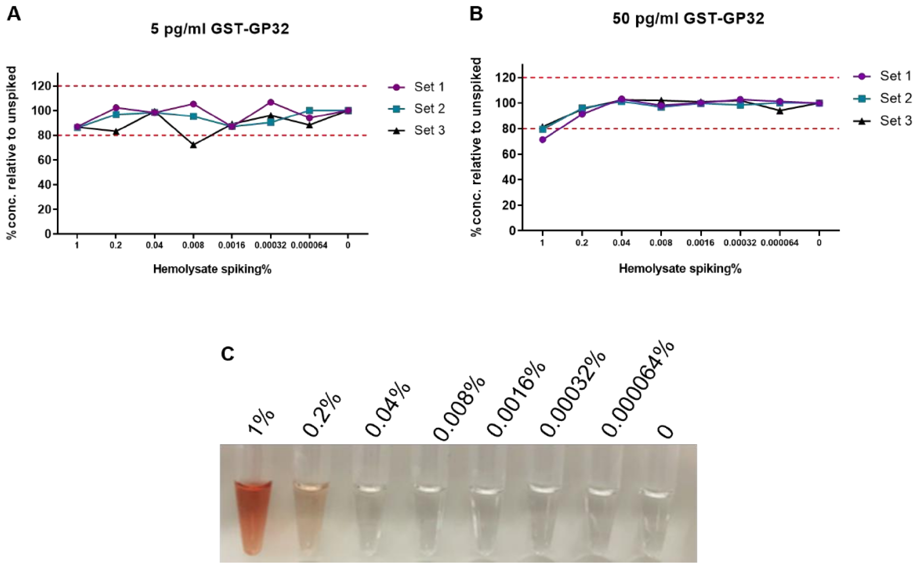

Supplementary figure 3. Haemoglobin interference. Control CSF was spiked with haemolysate and serial diluted to give range of equivalent % haemolysate. CSF was also spiked with either 5 pg/ml (A) or 50 pg/ml GST-GP32 (B) and poly(GP) concentration measured using Simoa assay. Three sets at each GST-GP32 concentration were assayed and % error in predicated concentration was plotted for each sample. Red dotted lines at +/- 20% from expected poly(GP) concentration. C) Visual appearance of CSF after haemolysate spiking.

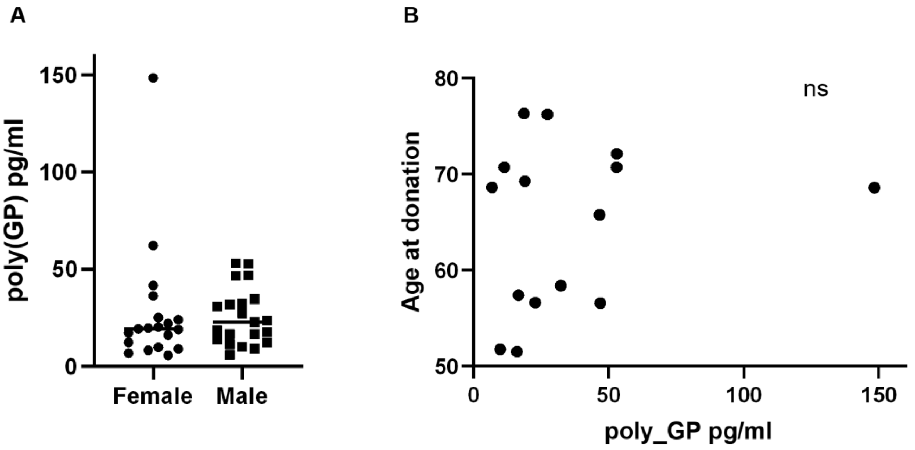

Supplementary Figure 4. Analysis of poly(GP) CSF levels with clinical features. A) No difference between female and male *C9orf72* expansion carriers in CSF poly(GP) levels. **B).** Age at visit for symptomatic *C9orf72* expansion carriers plotted against CSF poly(GP) levels.

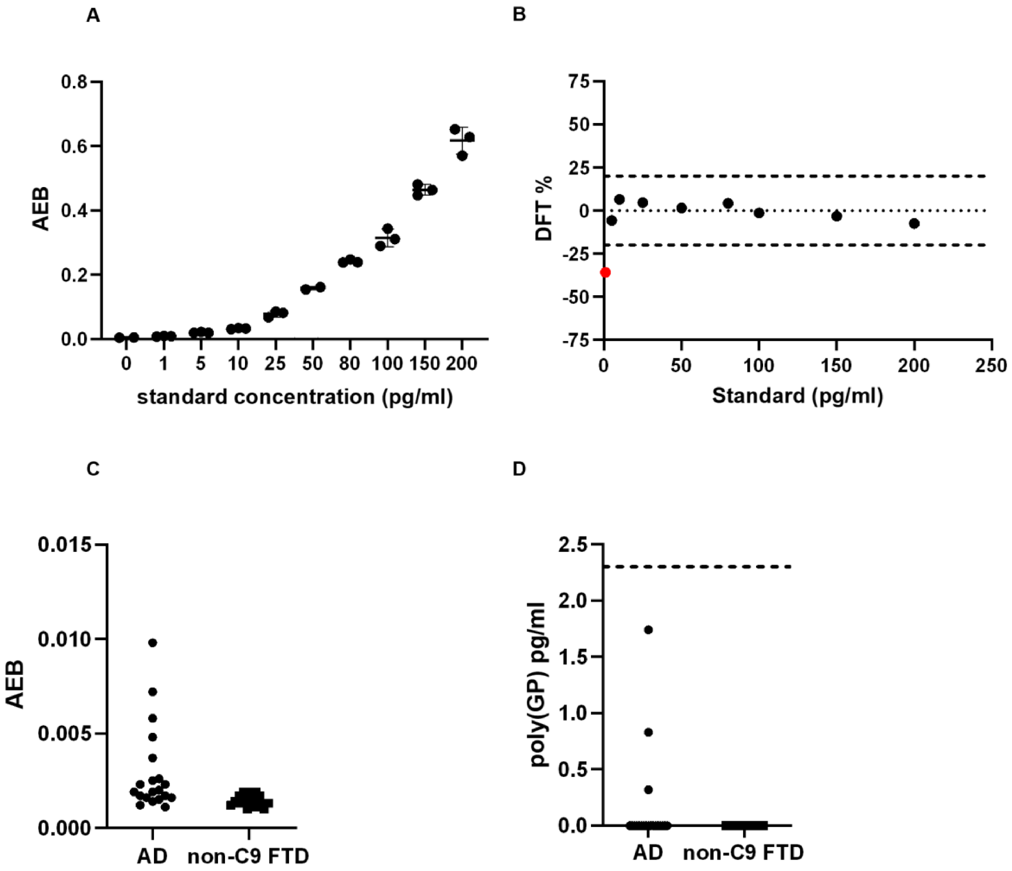

Supplementary Figure 5. Assessment of Alzheimer's disease and non-*C9orf72* expansion FTD patient CSF. In order to run control neurodegenerative disease patient CSF, additional GP57*-60* antibody was coupled to Simoa beads. Using this newly generated capture reagent, CSF from 20 Alzheimer's disease and 20 non-*C9orf72* expansion FTD carriers (12 nonfluent variant primary progressive aphasia and 8 behavioural variant FTD) were assessed for poly(GP) levels. Standard calibrators were run at the same time to assess the assay performance. **A)** AEB values from poly(GP) standards run in triplicate (mean and std). **B)** The difference from total (DFT) calculated for each standard for this assay. DFT = % difference between predicted concentration and actual concentration of calibrators. Dotted lines at +/- 20% acceptance level. The 1 pg/ml standard had a DFT of -36%. **C)** Raw AEB values for each sample, each dot represents the mean of duplicate measures. **D)** Predicted poly(GP) pg/ml values for each sample based on the standard calibrators used for this assay. 37/40 samples had AEB values too low to quantify. The dotted line (at 2.3 pg/ml) is the LLOQ predicted for this assay using the Quanterix assay developer tool.

Wilson KM, *et al. J Neurol Neurosurg Psychiatry* 2022; 93:761–771. doi: 10.1136/jnnp-2021-328710

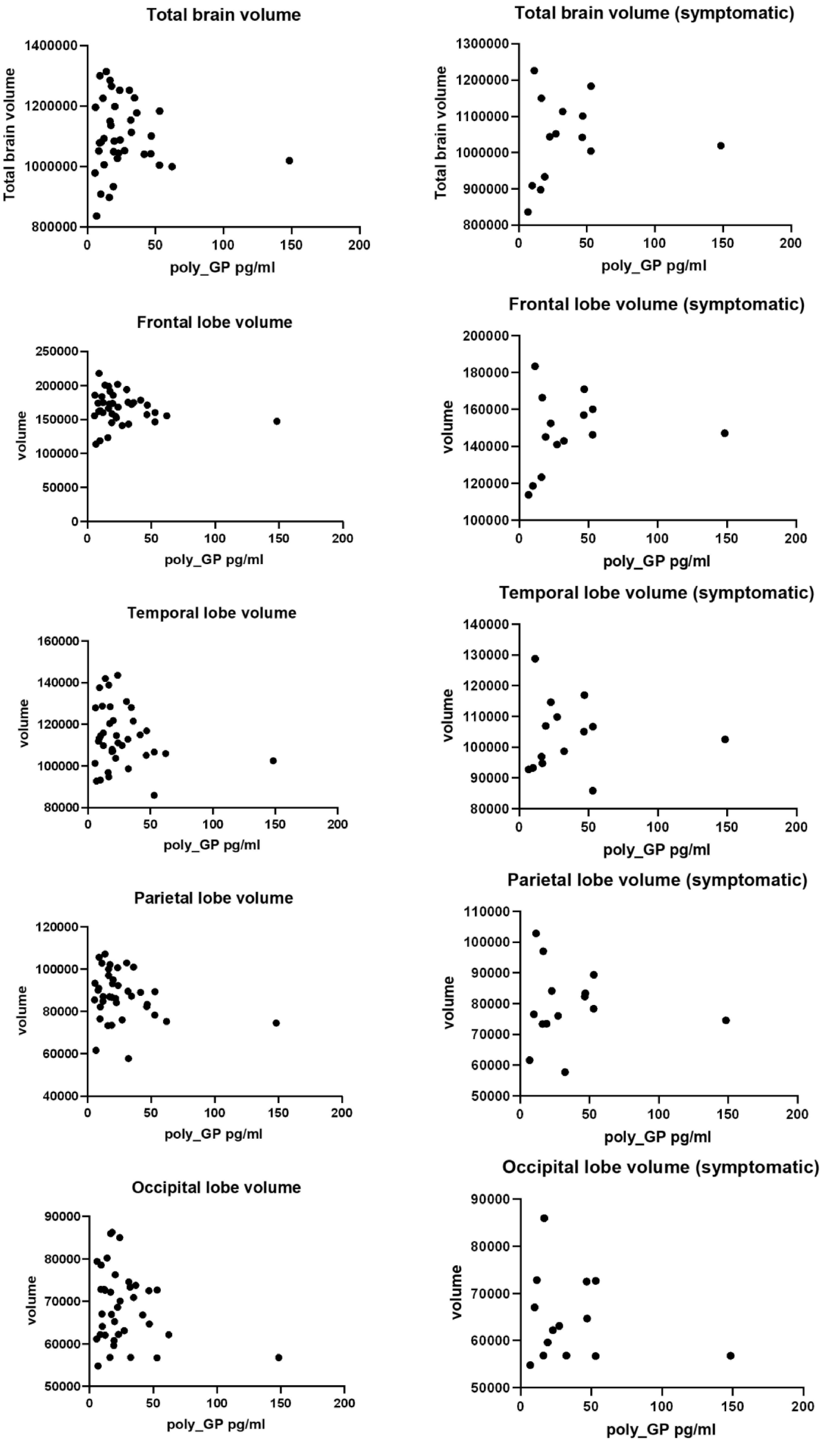
Total brain volume
Total brain volume
poly_GP pg/ml
Total brain volume (symptomatic)
Total brain volume
poly_GP pg/ml
Frontal lobe volume
volume
poly_GP pg/ml
Frontal lobe volume (symptomatic)
volume
poly_GP pg/ml
Temporal lobe volume
volume
poly_GP pg/ml
Temporal lobe volume (symptomatic)
volume
poly_GP pg/ml
Parietal lobe volume
volume
poly_GP pg/ml
Parietal lobe volume (symptomatic)
volume
poly_GP pg/ml
Occipital lobe volume
volume
poly_GP pg/ml
Occipital lobe volume (symptomatic)
volume
poly_GP pg/ml

Supplementary Figure 6. Analysis of brain volume with poly(GP) CSF levels. Left-hand side; total brain volume, temporal lobe, parietal lobe, occipital lobe and frontal lobe volumes against poly(GP) CSF levels from *C9orf72* expansion carriers (N=38). Right-hand side analysis of same regions from symptomatic *C9orf72* expansion carriers (N=14).

Wilson KM, *et al. J Neurol Neurosurg Psychiatry* 2022; 93:761–771. doi: 10.1136/jnnp-2021-328710

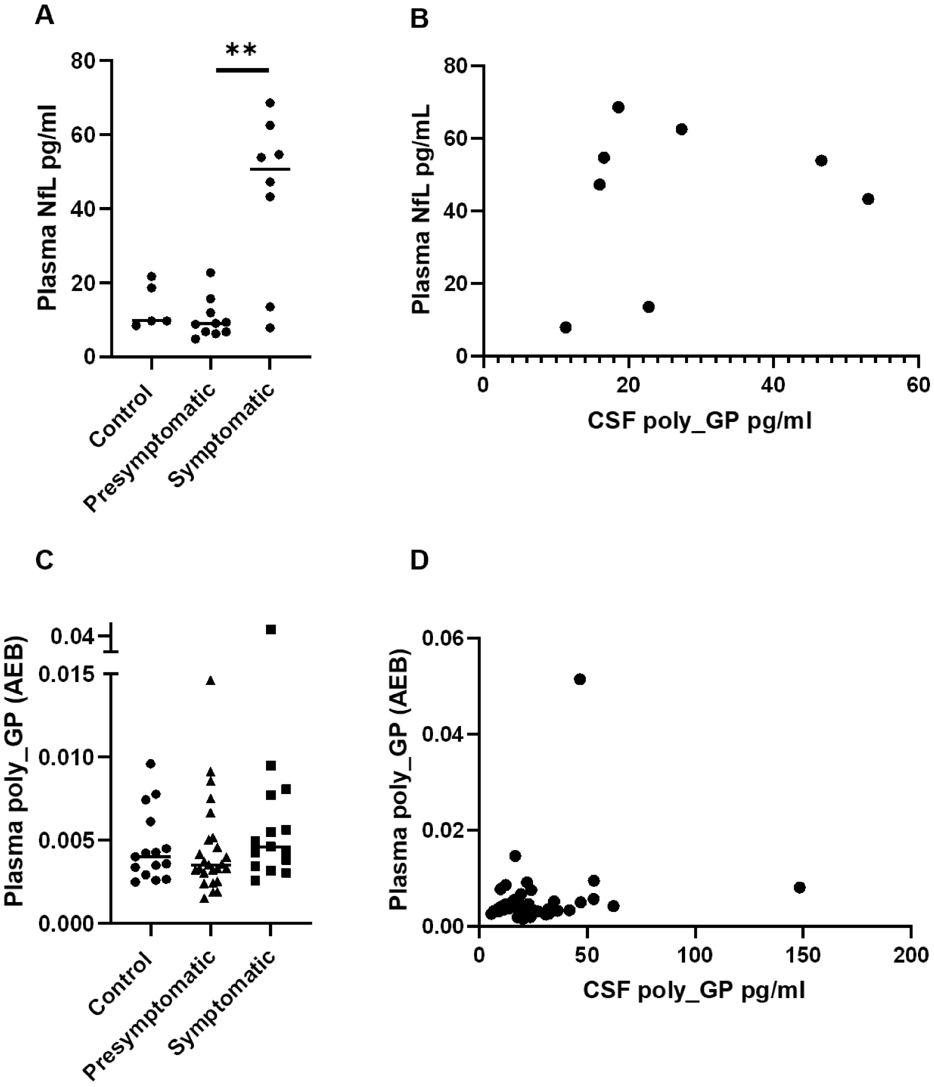

Supplementary Figure 7. Analysis of plasma biomarkers from matched CSF donors. Plasma samples from 5 controls, 10 presymptomatic and 8 symptomatic *C9orf72* expansion carriers whom also had poly(GP) CSF measured. **A)** Plasma NfL levels were significantly higher in symptomatic carriers compared to presymtomatic carriers (Kruskal Wallis and Dunn's multiple comparisons, ** p<0.01). **B)** No correlation was observed between plasma NfL levels and CSF poly(GP) levels in the available matched samples from 8 symptomatic cases. **C)** Raw AEB signals from Simoa assay optimised to measure poly(GP) in plasma. No difference was observed between controls or *C9orf72* expansion carriers. **D)** Raw AEB signals from plasma samples plotted against matched samples CSF poly(GP) levels.

	Avg. from 3 independent assays		Comp. TEST4 to average		Comp. TEST5 to average		Comp. TEST6 to average		Comp. TEST7 to average	
Calibration curve	Analyst 1		Analyst 2		Analyst 2		Analyst 1		Analyst 2	
pg/ml	Mean AEB	CV%	AEB	CV% comp.	AEB	CV% comp.	AEB	CV% comp.	AEB	CV% comp.
200	0.8360	7%	0.7042	12%	1.0470	16%	0.7481	8%	0.7647	6%
150	0.6185	7%	0.5497	8%	0.7831	17%	0.5882	4%	0.5689	6%
100	0.4337	9%	0.3752	10%	0.5382	15%	0.3729	11%	0.4064	5%
80	0.3400	7%	0.2831	13%	0.4256	16%	0.3012	9%	0.3000	9%
50	0.2182	5%	0.1848	12%	0.2682	15%	0.2003	6%	0.1957	8%
25	0.1117	4%	0.0992	8%	0.1381	15%	0.1009	7%	0.1101	1%
10	0.0453	6%	0.0421	5%	0.0597	19%	0.0458	1%	0.0432	3%
5	0.0255	13%	0.0212	13%	0.0287	8%	0.0205	15%	0.0236	5%
1	0.0058	12%	0.0054	5%	0.0083	25%	0.0062	5%	0.0073	16%
0	0.0022	22%	0.0020	7%	0.0026	11%	0.0021	3%	0.0023	1%

Calibration curve	Test 1	Test 2	Test 3	Test 4	Test 5	Test 6	Test 7
pg/ml	DFT %	DFT %	DFT %	DFT %	DFT %	DFT %	DFT %
200	-4.48	-6.03	-1.56	-1.07	-0.32	-0.05	-1.85
150	1.44	2.77	1.23	-2.12	0.82	-3.53	0.38
100	-0.95	0.16	0.34	-1.35	-1.23	3.40	-5.00
80	3.60	4.65	0.91	5.86	0.51	3.15	4.57
50	4.43	1.58	-1.30	3.23	0.78	-1.78	2.67
25	2.17	-1.64	-0.12	-2.33	-0.33	-0.48	-5.80
10	-2.88	1.03	-0.78	-5.98	-4.75	-10.31	3.83
5	-23.87	-14.18	4.63	-2.05	5.25	7.46	2.53
1	17.80	11.03	-3.91	6.68	-2.64	-1.79	-6.40
0	NaN	NaN	NaN	NaN	NaN	NaN	NaN

Supplementary table 1 and 2) Standard curve CV% and DFT% assessment.

CV% calculated from average AEB values from 3 initial standard curves. Total of 7 assays carried out by 2 independent analysts. DFT = difference from total % predicted concentration of standards (pg/ml) versus actual.

	Mean for 3 set of QCs on		Comp. TEST4 to average		Comp. TEST5 to average		Comp. TEST6 to average		Comp. TEST7 to average	
QCs	Mean AEB	CV%	Mean AEB	CV% comp.	Mean AEB	CV% comp.	Mean AEB	CV% comp.	Mean AEB	CV% comp.
HQC 140pg/ml	0.5540	14%	0.4940	8%	0.7309	19%	0.5160	5%	0.4856	9%
MQC 75pg/ml	0.3066	8%	0.2714	9%	0.4197	22%	0.2752	8%	0.3062	0%
LQC 15pg/ml	0.0610	6%	0.0526	10%	0.0888	26%	0.0568	5%	0.0651	5%

Supplementary table 3) Assessment of quality control samples (QCs) CV%.

	Test 1	Test 2	Test 3	Test 4	Test 5	Test 6	Test 7
QCs	DFT %	DFT %	DFT %	DFT %	DFT %	DFT %	DFT %
HQC 140pg/ml	0%	9%	12%	3%	1%	3%	10%
MQC 75pg/ml	7%	9%	6%	4%	-5%	22%	-4%
LQC 15pg/ml	8%	10%	9%	11%	-6%	8%	0%

Supplementary table 4) Assessment of quality control samples (QCs) DFT%.

Intraplate variability	Position 1		Position 2		Position 3		Average of 3 sets	
QCs	Mean AEB	CV%	Mean AEB	CV%	Mean AEB	CV%	Mean AEB	Total CV%
HQC 140pg/ml	0.6394	6%	0.6058	1%	0.6149	2%	0.6201	3%
MQC 75pg/ml	0.3342	2%	0.3305	0%	0.3125	2%	0.3257	4%
LQC 15pg/ml	0.0651	1%	0.0605	3%	0.0618	1%	0.0625	4%

Supplementary table 5) Intraplate variability assessment of CV%.

Wilson KM, *et al. J Neurol Neurosurg Psychiatry* 2022; 93:761–771. doi: 10.1136/jnnp-2021-328710

Preparation variability	Analyst 1								Analyst 2									
	Prep. 1		Prep. 2		Prep. 3		Assay Mean		Prep. 1		Prep. 2		Prep. 3		Assay Mean		Total Mean	Total CV
QCs	Mean AEB	CV%	Mean AEB	CV%	Mean AEB	CV%	Mean AEB	CV%	Mean AEB	CV%	Mean AEB	CV%	Mean AEB	CV%	Mean AEB	CV%	Mean AEB	CV%
HQC 140pg/ml	0.5160	1%	0.5140	3%	0.5180	2%	0.5160	0%	0.4940	1%	0.4814	2%	0.4675	2%	0.4930	2%	0.4985	4%
MQC 75pg/ml	0.2752	NaN	0.2835	3%	0.2852	9%	0.2813	2%	0.2714	1%	0.2831	2%	0.2666	6%	0.2876	0%	0.2775	3%
LQC 15pg/ml	0.0568	3%	NaN	14%	0.0604	2%	0.0586	4%	0.0526	3%	0.0540	12%	0.0526	5%	0.0594	11%	0.0553	6%

Supplementary table 6) Reproducibility assessment using independently prepared QCs.

Matrix control: QC4	Test 1	Test 2	Test 3	Test 4	CV%
Mean AEB	0.0526	0.0576	0.0696	0.0553	13%
Predicted concentration pg/ml	26.7	28.1	24.6	25.2	6%

Supplementary table 7) Reproducibility of matrix control CV%.

	neat - anchore	1:2			1:4			1:8			1:16		
	**Mean conc.**	**Mean conc.**	**Predicted neat**	Error%	**Mean conc.**	**Predicted neat**	Error%	**Mean conc.**	**Predicted neat**	Error%	**Mean conc.**	**Predicted neat**	Error%
**S1**	15.16	13.76	27.5	-82%	7.40	29.6	-95%	4.39	35.2	-132%	2.36	37.7	-149%
**S2**	11.75	9.96	19.9	-70%	5.66	22.6	-92%	3.19	25.5	-117%	1.37	21.8	-86%
**S3**	11.68	9.58	19.2	-64%	6.68	26.7	-129%	3.21	25.7	-120%	1.59	38.8	-232%
**S4**	21.84	17.63	35.3	-61%	11.68	46.7	-114%	6.16	49.3	-126%	2.70	43.2	-98%
**S5**	11.68	9.08	18.2	-55%	5.78	23.1	-98%	3.22	25.8	-121%	1.03	25.3	-117%
**S6**	8.62	9.41	18.8	-118%	5.67	22.7	-163%	3.19	25.5	-196%	1.21	29.6	-243%
**Average:**				-75%			-115%			-135%			-154%

	1:2 - anchore	1:4			1:8			1:16		
	**Mean conc.**	**Mean conc.**	**Predicted 1:2**	Error%	**Mean conc.**	**Predicted 1:2**	Error%	**Mean conc.**	**Predicted 1:2**	Error%
**S1**	13.76	7.40	14.8	7%	4.39	17.6	-28%	2.36	18.9	-37%
**S2**	9.96	5.66	11.3	-14%	3.19	12.8	-28%	1.37	10.9	-10%
**S3**	9.58	6.68	13.4	-39%	3.21	12.8	-34%	1.59	19.4	-102%
**S4**	17.63	11.68	23.4	-32%	6.16	24.6	-40%	2.70	21.6	-22%
**S5**	9.08	5.78	11.6	-27%	3.22	12.9	-42%	1.03	12.6	-39%
**S6**	9.41	5.67	11.3	-21%	3.19	12.8	-36%	1.21	14.8	-57%
**Average:**				-21%			-35%			-45%

Supplementary table 8) Dilutional parallelism was assessed by running CSF from six C9orf72 expansion positive donors either neat, 1:2, 1:4, 1:8 and 1:16 in diluent A**.**

QC4	Meas. 1	Meas. 2	Mean AEB	CV%	Pred. conc. (pg/ml)	CV% for mean AEB	CV% for pred. conc (pg/ml)
Fresh	0.0737	0.0654	0.0696	8%	24.6	4%	5%
Freeze-thaw 1	0.0805	0.0691	0.0748	11%	26.5		
Freeze-thaw 2	0.0732	0.0769	0.0751	4%	26.6		
Freeze-thaw 3	0.0698	0.0685	0.0691	1%	24.4		

Supplementary table 9) Freeze-thaw stability of poly(GP) from human CSF.

pg/ml	Mean AEB				CV% fresh vs. F-T 1	CV% fresh vs. F-T 2	CV% fresh vs. F-T 3	% Error in prediction concentration			
	Fresh	Freeze-thaw 1	Freeze-thaw 2	Freeze-thaw 3				Fresh	Freeze-thaw 1	Freeze-thaw 2	Freeze-thaw 3
200	0.7481	0.7675	0.7258	0.7303	2%	2%	2%	-0.05	-1.22	-3.51	-11.97
150	0.5882	0.5773	0.5568	0.5373	1%	4%	6%	-3.53	1.43	-0.01	-3.75
100	0.3729	0.3964	0.3822	0.3662	4%	2%	1%	3.40	1.42	2.00	-0.74
80	0.3012	0.3280	0.3003	0.3001	6%	0%	0%	3.15	-0.75	5.55	-1.05
50	0.2003	0.2067	0.2086	0.1846	2%	3%	6%	-1.78	0.89	-3.28	4.52
25	0.1009	0.1167	0.1004	0.0978	10%	0%	2%	-0.48	-9.09	1.52	2.87
10	0.0458	0.0426	0.0456	0.0375	5%	0%	14%	-10.31	5.27	-11.82	12.17
5	0.0205	0.0231	0.0200	0.0215	8%	2%	3%	7.46	2.23	4.53	3.90
1	0.0062	0.0064	0.0054	0.0072	2%	10%	10%	-1.79	-3.95	2.52	-34.34
0	0.0021	0.0018	0.0020	0.0013	14%	4%	33%	NaN	NaN	NaN	NaN

Supplementary table 10) Freeze-thaw stability of GST-GP32 standard.

Wilson KM, *et al. J Neurol Neurosurg Psychiatry* 2022; 93:761–771. doi: 10.1136/jnnp-2021-328710

				**C9orf72 Expansion Carriers**	
	**Controls**	**Alzheimer's disease**	**Non-*C9orf72* FTD**	**Presymptomatic**	**Symptomatic**
N	15	20	20	26	17
**Age at CSF**	48.2 (11.2)	61.2 (5.6)	65.6 (8.9)	40.3 (10.5)	64.1 (8.2)
**Sex (% female)**	86.7	45	25.0	57.7	41.2
**MMSE (/30)**	29.1 (1.5)	20.0 (5.5)	24.2 (5.0)	29.7 (0.8)	22.8 (6.3)

Supplementary Table 11) Demographics of cases used in the study. *N*, number of participants. Values are shown as mean (standard deviation).

Appendix

List of GENFI consortium authors:

Sónia Afonso[1], Maria Rosario Almeida[2], Sarah Anderl-Straub[3], Christin Andersson[4], Anna Antonell[5], Silvana Archetti[6], Andrea Arighi[7], Mircea Balasa[8], Myriam Barandiaran[9], Nuria Bargalló[10], Robart Bartha[11], Benjamin Bender[12], Alberto Benussi[13], Maxime Bertoux[14,15], Anne Bertrand[16,17], Valentina Bessi[19], Sandra Black[20], Martina Bocchetta[21], Sergi Borrego-Ecija[8], Jose Bras[22], Alexis Brice[16], Rose Bruffaerts[23,24], Agnès Camuzat[8], Marta Cañada[25], Valentina Cantoni[13], Paola Caroppo[26], David Cash[21], Miguel Castelo-Branco[2], Olivier Colliot[16,17] , Rhian Convery[13], Thomas Cope[27], Adrian Danek[18], Vincent Deramecourt[29,30,31], Giuseppe Di Fede[26], Alina Díez[31], Diana Duro[2], Chiara Fenoglio[7], Camilla Ferrari[32], Catarina B. Ferreira[33], Nick Fox[21], Morris Freedman[34], Giorgio Fumagalli[7], Aurélie Funkiewiez[16,35], Alazne Gabilondo[31], Roberto Gasparotti[37], Serge Gauthier[38], Stefano Gazzina[39], Giorgio Giaccone[26], Ana Gorostidi[36], Lisa Graf[40], Caroline Greaves[21], Rita Guerreiro[22], Tobias Hoegen[41], Begoña Indakoetxea[9], Vesna Jelic[42], Lize Jiskoot[43], Ron Keren[44], Gregory Kuchcinski[27,28,29] , Tobias Langheinrich[45,46], Isabelle Le Ber[16,35,47], Thibaud Lebouvier[27,28,29], Maria João Leitão[48], Johannes Levin[,18,19,50], Albert Lladó[8], Gemma Lombardi[32], Jolina Lombardi[3], Sandra Loosli[51], Carolina Maruta[52], Simon Mead[53], Gabriel Miltenberger[2], Rick van Minkelen[54], Sara Mitchell[20], Fermin Moreno[9], Benedetta Nacmias[32,55], Annabel Nelson[21], Jennifer Nicholas[56], Linn Öijerstedt[57,58], Janne M. Papma[43], Florence Pasquier[14,15], Georgia Peakman[21], Yolande Pijnenburg[59], Cristina Polito[60], Enrico Premi[61], Sara Prioni[26], Catharina Prix[51], Veronica Redaelli[26], Daisy Rinaldi[16,35,47], Tim Rittman[27], Ekaterina Rogaeva[62], Pedro Rosa-Neto[63], Giacomina Rossi[26], Martin Rossor[21], Isabel Santana[64], Beatriz Santiago[26], Dario Saracino[16,35,47], Elio Scarpini[7], Sonja Schönecker[51], Rachelle Shafei[21], Christen Shoesmith[65], Sandro Sorbi[32,55], Miguel Tábuas-Pereira[66], Fabrizio Tagliavini[26], Mikel Tainta[31], Ricardo Taipa[67], David Tang-Wai[68], David L Thomas[69], Paul Thompson[70], Carolyn Timberlake[27], Pietro Tiraboschi[26], Emily Todd[21], Philip Van Damme[71], Mathieu Vandenbulcke[72], Ana Verdelho[73], Jorge Villanua[74], Jason Warren[21], Carlo Wilke[40], Elisabeth Wlasich[41], Miren Zulaica[36]

List of GENFI consortium affiliations:

1. Instituto Ciencias Nucleares Aplicadas a Saude, Universidade de Coimbra, Coimbra, Portugal.
2. Faculty of Medicine, University of Coimbra, Coimbra, Portugal.
3. Department of Neurology, University of Ulm, Ulm, Germany.
4. Department of Clinical Neuroscience, Karolinska Institutet, Stockholm, Sweden.
5. Alzheimer's disease and Other Cognitive Disorders Unit, Neurology Service, Hospital Clínic, Barcelona, Spain.
6. Biotechnology Laboratory, Department of Diagnostics, ASST Brescia Hospital, Brescia, Italy.
7. Fondazione IRCCS Ca' Granda Ospedale Maggiore Policlinico, Neurodegenerative Diseases Unit, Milan, Italy; University of Milan, Centro Dino Ferrari, Milan, Italy.
8. Alzheimer's disease and Other Cognitive Disorders Unit, Neurology Service, Hospital Clínic, Barcelona, Spain.
9. Cognitive Disorders Unit, Department of Neurology, Donostia University Hospital, San Sebastian, Gipuzkoa, Spain; Neuroscience Area, Biodonostia Health Research Insitute, San Sebastian, Gipuzkoa, Spain.
10. Imaging Diagnostic Center, Hospital Clínic, Barcelona, Spain.
11. Department of Medical Biophysics, The University of Western Ontario, London, Ontario, Canada; Centre for Functional and Metabolic Mapping, Robarts Research Institute, The University of Western Ontario, London, Ontario, Canada.
12. Department of Diagnostic and Interventional Neuroradiology, University of Tübingen, Tübingen, Germany.
13. Centre for Neurodegenerative Disorders, Department of Clinical and Experimental Sciences, University of Brescia, Italy.
14. Inserm 1172, Lille, France.
15. CHU, CNR-MAJ, Labex Distalz, LiCEND Lille, France.
16. Sorbonne Université, Paris Brain Institute – Institut du Cerveau – ICM, Inserm U1127, CNRS UMR 7225, AP-HP - Hôpital Pitié-Salpêtrière, Paris, France.
17. Centre pour l'Acquisition et le Traitement des Images, Institut du Cerveau et la Moelle, Paris, France.
18. Department of Neurology, Ludwig-Maximilians Universität München, Munich, Germany.
19. Department of Neuroscience, Psychology, Drug Research and Child Health, University of Florence, Florence, Italy.
20. Sunnybrook Health Sciences Centre, Sunnybrook Research Institute, University of Toronto, Toronto, Canada.
21. Dementia Research Centre, Department of Neurodegenerative Disease, UCL Institute of Neurology, Queen Square, London, UK.
22. Center for Neurodegenerative Science, Van Andel Institute, Grand Rapids, Michigan, MI 49503, USA.
23. Laboratory for Cognitive Neurology, Department of Neurosciences, KU Leuven, Leuven, Belgium.
24. Biomedical Research Institute, Hasselt University, 3500 Hasselt, Belgium.
25. CITA Alzheimer, San Sebastian, Gipuzkoa, Spain.
26. Fondazione IRCCS Istituto Neurologico Carlo Besta, Milano, Italy.
27. Department of Clinical Neuroscience, University of Cambridge, Cambridge, UK.

28. Univ Lille, France.
29. Inserm 1172, Lille, France.
30. CHU, CNR-MAJ, Labex Distalz, LiCEND Lille, France.
31. Neuroscience Area, Biodonostia Health Research Insitute, San Sebastian, Gipuzkoa, Spain.
32. Department of Neuroscience, Psychology, Drug Research and Child Health, University of Florence, Florence, Italy.
33. Laboratory of Neurosciences, Institute of Molecular Medicine, Faculty of Medicine, University of Lisbon, Lisbon, Portugal.
34. Baycrest Health Sciences, Rotman Research Institute, University of Toronto, Toronto, Canada
35. Centre de référence des démences rares ou précoces, IM2A, Département de Neurologie, AP-HP - Hôpital Pitié-Salpêtrière, Paris, France
36. Neuroscience Area, Biodonostia Health Research Insitute, San Sebastian, Gipuzkoa, Spain.
37. Neuroradiology Unit, University of Brescia, Brescia, Italy.
38. Alzheimer Disease Research Unit, McGill Centre for Studies in Aging, Department of Neurology & Neurosurgery, McGill University, Montreal, Québec, Canada.
39. Neurology, ASST Brescia Hospital, Brescia, Italy.
40. Department of Neurodegenerative Diseases, Hertie-Institute for Clinical Brain Research and Center of Neurology, University of Tübingen, Tübingen, Germany.
41. Neurologische Klinik, Ludwig-Maximilians-Universität München, Munich, Germany.
42. Division of Clinical Geriatrics, Karolinska Institutet, Stockholm, Sweden.
43. Department of Neurology, Erasmus Medical Center, Rotterdam, Netherlands.
44. The University Health Network, Toronto Rehabilitation Institute, Toronto, Canada.
45. Division of Neuroscience and Experimental Psychology, Wolfson Molecular Imaging Centre, University of Manchester, Manchester, UK .
46. Manchester Centre for Clinical Neurosciences, Department of Neurology, Salford Royal NHS Foundation Trust, Manchester, UK.
47. Département de Neurologie, AP-HP - Hôpital Pitié-Salpêtrière, Paris, France
48. Centre of Neurosciences and Cell Biology, Universidade de Coimbra, Coimbra, Portugal.
49. German Center for Neurodegenerative Diseases (DZNE), Munich, Germany.
50. Munich Cluster of Systems Neurology (SyNergy), Munich, Germany.
51. Neurologische Klinik, Ludwig-Maximilians-Universität München, Munich, Germany.
52. Laboratory of Language Research, Centro de Estudos Egas Moniz, Faculty of Medicine, University of Lisbon, Lisbon, Portugal.
53. MRC Prion Unit, Department of Neurodegenerative Disease, UCL Institute of Neurology, Queen Square, London, UK.
54. Department of Clinical Genetics, Erasmus Medical Center, Rotterdam, Netherlands.
55. IRCCS Fondazione Don Carlo Gnocchi, Florence, Italy.
56. Department of Medical Statistics, London School of Hygiene and Tropical Medicine, London, UK.
57. Center for Alzheimer Research, Division of Neurogeriatrics, Department of Neurobiology, Care Sciences and Society, Bioclinicum, Karolinska Institutet, Solna, Sweden.
58. Unit for Hereditary Dementias, Theme Aging, Karolinska University Hospital, Solna, Sweden.
59. Amsterdam University Medical Centre, Amsterdam VUmc, Amsterdam, Netherlands.
60. Department of Biomedical, Experimental and Clinical Sciences “Mario Serio”, Nuclear Medicine Unit, University of Florence, Florence, Italy.
61. Stroke Unit, ASST Brescia Hospital, Brescia, Italy.
62. Tanz Centre for Research in Neurodegenerative Diseases, University of Toronto, Toronto, Canada
63. Translational Neuroimaging Laboratory, McGill Centre for Studies in Aging, McGill University, Montreal, Québec, Canada.
64. University Hospital of Coimbra (HUC), Neurology Service, Faculty of Medicine, University of Coimbra, Coimbra, Portugal; Center for Neuroscience and Cell Biology, Faculty of Medicine, University of Coimbra, Coimbra, Portugal.
65. Department of Clinical Neurological Sciences, University of Western Ontario, London, Ontario, Canada.
66. Neurology Department, Centro Hospitalar e Universitario de Coimbra, Coimbra, Portugal.
67. Neuropathology Unit and Department of Neurology, Centro Hospitalar do Porto - Hospital de Santo António, Oporto, Portugal.
68. The University Health Network, Krembil Research Institute, Toronto, Canada.
69. Neuroimaging Analysis Centre, Department of Brain Repair and Rehabilitation, UCL Institute of Neurology, Queen Square, London, UK.

70. Division of Neuroscience and Experimental Psychology, Wolfson Molecular Imaging Centre, University of Manchester, Manchester, UK.
71. Neurology Service, University Hospitals Leuven, Belgium; Laboratory for Neurobiology, VIB-KU Leuven Centre for Brain Research, Leuven, Belgium.
72. Geriatric Psychiatry Service, University Hospitals Leuven, Belgium; Neuropsychiatry, Department of Neurosciences, KU Leuven, Leuven, Belgium.
73. Department of Neurosciences and Mental Health, Centro Hospitalar Lisboa Norte - Hospital de Santa Maria & Faculty of Medicine, University of Lisbon, Lisbon, Portugal.
74. OSATEK, University of Donostia, San Sebastian, Gipuzkoa, Spain.

BMJ Publishing Group Limited (BMJ) disclaims all liability and responsibility arising from any reliance placed on this supplemental material which has been supplied by the author(s)

UK Dementia Research Institute	Dementia Research Institute at UCL, Cruciform Building		UCL
Program: C9ORF72	**Standard Operating Procedure**		Page 1 of 6
Title:	**Simoa Homebrew poly(GP) assay protocol**		
Protocol Id:	**mGP/GP57-60_HD-X**	Protocol Version No.:	**2**
Responsible Scientist:	Eszter Katona	Date last revised:	**28/01/2022**
Approvers:	Adrian Isaacs and Amanda Heslegrave	Approval Date:	**16/02/2022**

Contents:

Step	Content
Table 1	Reagents
Table 2	Consumables
Table 3	Reagent volumes
Table 4	Sample volumes
Table 5	Assay set-up
Table 6	Procedure 1 – Maintenance task before a run
Table 7	Procedure 2 – Running assay on the HD-X
Table 8	Procedure 3 – After run: Data extraction and post-run maintenance

Table 1: Reagents

	Reagent Name	Reagent Concentration	Reagent Source	Catalogue Number
1.	System Wash Buffer 1	1x	Quanterix	100486
2.	System Wash Buffer 2	1x	Quanterix	100487
3.	Bead Diluent	1x	Quanterix	100458
4.	SBG Diluent	1x	Quanterix	100376
5.	Homebrew Detector/Sample Diluent	1x	Quanterix	101359
6.	Sample Diluent A kit	1x	Quanterix	101575
7.	SBG Concentrate	dependent on lot#	Quanterix	103397
8.	RGP Reagent	1x	Quanterix	103159
9.	Homebrew Helper Beads	dependent on lot#	Quanterix	103208
10.	Homebrew Carboxylated Beads	dependent on lot#	Quanterix	103207
11.	Standard peptide (GST-GP32)	Two starting stocks: 15000, 1500 pg/ml	Custom made – Wave Life Sciences	N/A
12.	Capture antibody (mGP conjugated to carboxylated beads)	Depends on aliquot	Developmental Studies Hybridoma Bank, Target ALS Foundation	tALS 828.179
13.	Detector antibody (biotinylated GP57-60)	Depends on aliquot	Custom made	N/A

UK Dementia Research Institute	**Dementia Research Institute at UCL, Cruciform Building**			UCL
Program: C9ORF72	**Standard Operating Procedure**		Page 2 of 6	
Title:	**Simoa Homebrew poly(GP) assay protocol**			
Protocol Id:	**mGP/GP57-60_HD-X**	Protocol Version No.:	**2**	
Responsible Scientist:	Eszter Katona	Date last revised:	**28/01/2022**	
Approvers:	Adrian Isaacs and Amanda Heslegrave	Approval Date:	**16/02/2022**	

Table 2: Consumables

	Name	Source	Catalogue Number
1.	Conical Well Plates for HD-1/X	Quanterix	101457
2.	Conductive Tips for Simoa HD-1/X Analyzer	Quanterix	101726
3.	Simoa Cuvettes Bulk Pack (rev 2)	Quanterix	103346
4.	Simoa Discs (16)	Quanterix	100001
5.	Reagent Bottle Pack for Simoa HD-1/X Analyzer	Quanterix	102411
6.	Simoa HD-1 Sealing Oil	Quanterix	100206
7.	**Alternative:** Disc Kit for Simoa HD-1/X Analyzer (rev 2)	Quanterix	103347
8.	DynaMag-2 Magnet magnetic separator	Thermo Fisher Scientific	12321D

Table 3: Reagent volumes

Reagent	Working concentration	Diluent	Volume /measurement (µl)	Extra pipettor volume/ measurement (µl)	Dead volume/ reagent holder (µl)	Total volume for n number of measurements (µl)
Assay beads (mGP)	6×10^6 beads/ml	Bead diluent	25 µl	10 µl	600 µl	n × (25 + 10) + 600
Helper beads	14×10^6 beads/ml					
Detector antibody	0.3 µg/ml	Homebrew Detector/ Sample diluent	20 µl			n × (20 + 10) + 600
SBG	50 pM	SBG diluent	100 µl			n × (100 + 10) + 600
RGP	N/A	N/A	50 µl			n × (50 + 10) + 600

Table 4: Sample volumes

Sample type	Working concentration	Diluent	Volume/ Measurement (µl)	Extra pipettor volume/measurement (µl)	Dead volume/well (µl)	Total volume/sample in duplicate measurements (µl)
CSF	1:2	Diluent A	100	10	30	2 × (100 + 10) + 30
Calibrator	200, 150, 100, 80, 50, 25, 10, 5, 1, 0 pg/ml		100	10		2 × (100 + 10) + 30

Wilson KM, *et al. J Neurol Neurosurg Psychiatry* 2022; 93:761–771. doi: 10.1136/jnnp-2021-328710

UK Dementia Research Institute	Dementia Research Institute at UCL, Cruciform Building		UCL
Program: C9ORF72	**Standard Operating Procedure**		Page 3 of 6
Title:	**Simoa Homebrew poly(GP) assay protocol**		
Protocol Id:	**mGP/GP57-60_HD-X**	Protocol Version No.:	**2**
Responsible Scientist:	Eszter Katona	Date last revised:	**28/01/2022**
Approvers:	Adrian Isaacs and Amanda Heslegrave	Approval Date:	**16/02/2022**

Table 5: Assay set-up

**Dilutions and Steps**	Dilution Description	Neat
	Steps	2-step assay neat 50 ul RGP
**Incubation time**	Step 1 (Beads, Sample, Detector)	47 x cadences (45s)
	Step 2 (SBG)	7 x cadences (45s)

Table 6: Procedure 1 – Maintenance task before a run

Step	Step Description
1.	If the first run of the day:<br>a) Turn on the PC and switch on the instrument.<br>b) Launch the SIMOA software and wait until the instrument initialises and the instrument status is 'Ready'.<br>c) Fill a reagent bottle with System Wash Buffer 1. Place the reagent bottle to 'Position 3' in a rack.<br>d) Go to 'Maintenance' tab and select 'Start of the day'. Click on 'Run task'.<br>e) Push rack into position 4 in the instrument and click 'Next' on the maintenance tab.<br>f) Wait for the run to finish and once it is done click 'Close' to close the maintenance tab. *Maintenance task takes ~ 20 min.*<br>g) Remove the rack from the instrument. *If 4 hrs have passed between the start of the assay run and the 'Start of the day' maintenance task, the 'Idle Fluid Prime' task needs to be performed before assay run can be initiated.*
2.	If not the first run of the day:<br>a) If > 4 hrs have passed since 'Start of the day' maintenance task or previous assay run, go to 'Maintenance' tab and select 'Idle Fluid Prime'.<br>b) Click 'Run task' and wait until maintenance task finishes. *Takes ~ 10 min.*<br>c) Once finished, click 'Close'.

Table 7: Procedure 2 – Running assay on the HD-X

Step	Step Description
1.	Once the maintenance task is finished, set up Homebrew assay, if not already done so.<br>a) You can download Homebrew assay definition from Quanterix Customer support website. This can be imported in the instrument at 'Custom Assay/ Assay Overview'.<br>b) To set up assay, go to 'Custom Assay' tab and select the name of the Homebrew assay.<br>c) Define number washes, dilution, assay beads, detector antibody, SBG, and RGP according to the conditions given above in the 'Assay set up' table.

Wilson KM, *et al. J Neurol Neurosurg Psychiatry* 2022; 93:761–771. doi: 10.1136/jnnp-2021-328710

UK Dementia Research Institute	Dementia Research Institute at UCL, Cruciform Building		UCL
Program: C9ORF72	**Standard Operating Procedure**		Page 4 of 6
Title:	**Simoa Homebrew poly(GP) assay protocol**		
Protocol Id:	**mGP/GP57-60_HD-X**	Protocol Version No.:	**2**
Responsible Scientist:	Eszter Katona	Date last revised:	**28/01/2022**
Approvers:	Adrian Isaacs and Amanda Heslegrave	Approval Date:	**16/02/2022**

	d) Set up calibrator concentrations and definitions under 'Plexes' tab. If helper beads are used, set up helper plex with the same calibrator concentrations. Set appropriate definitions and make sure that results are set to be hidden.
2.	Put required volume of RGP to a shaker at 800 rpm either at 30°C for ≥30 minutes or 25°C for ≥1 hr.
3.	Preparation of the assay bead:<br>a) Take out Bead Diluent on ice.<br>b) Add 1 ml Bead Diluent to a 2ml Eppendorf tube.<br>c) Add the required volume of Bead Diluent to a reagent bottle.<br>d) Vortex assay beads at high speed for 30 sec and add required volume to the 2 ml Eppendorf tube with 1 ml Bead Diluent.<br>e) If helper beads are used, perform the same procedure with the helper beads. Note, that both the assay and helper beads go into the same 2 ml Eppendorf tube containing the bead diluent.<br>f) Vortex mix for 5 sec and spin down for 10 sec at 1000×g.<br>g) Place the tube on a magnetic separator for 1 min.<br>h) Aspirate Bead Diluent then remove the tube from the magnetic separator.<br>i) Resuspend beads in 1 ml Bead Diluent from the reagent bottle, then vortex beads for 5 sec and spin it down briefly to make sure that all liquid is removed from the cap.<br>j) Add suspension to the reagent bottle. Pipette up and down twice to make sure all beads are added.<br>k) Close reagent bottle, label with Homebrew Bead barcode, and place on a rotator to keep beads in solution while the rest of the reagents are being prepared.
4.	Preparation of the detector antibody:<br>a) Take out Homebrew Detector/Sample Diluent and detector antibody stock on ice.<br>b) Add the required volume of Homebrew Detector/Sample Diluent for detector antibody dilution to a reagent holder.<br>c) Flick detector antibody stock tube to mix. Add the required volume of stock detector antibody to the diluent in the reagent holder.<br>d) Close reagent holder, label with Homebrew Detector barcode, and place on a rotator while preparing the rest of the reagents.
5.	Preparation of SBG:<br>a) Take out SBG diluent and SBG concentrate on ice.<br>b) Add the required volume of SBG diluent to a reagent holder.<br>c) Vortex SBG concentrate and add required volume to the diluent in the reagent holder to achieve 50 pM working concentration.<br>d) Close the reagent holder, label with Homebrew SBG barcode, and put on a rotator to mix.
6.	Preparation of the calibrators:<br>a) Take out Diluent A on ice.<br>b) Take out standard stock to ice, briefly vortex, and spin down.<br>c) Make up calibrator curve from peptide stock by serial dilution in Eppendorf tubes. Between each dilution, vortex dilution for 15 sec then briefly spin it down. Calibrator concentrations 200, 150, 100, 80, and 50 pg/ml are made up from the 15000 pg/ml stock while 25, 10, 5, 1, and 0 pg/ml are made up from the 1500 pg/ml stock.<br>d) Place ready calibrators on ice.

UK Dementia Research Institute	Dementia Research Institute at UCL, Cruciform Building		UCL
Program: C9ORF72	**Standard Operating Procedure**		Page 5 of 6
Title:	**Simoa Homebrew poly(GP) assay protocol**		
Protocol Id:	**mGP/GP57-60_HD-X**	Protocol Version No.:	**2**
Responsible Scientist:	Eszter Katona	Date last revised:	**28/01/2022**
Approvers:	Adrian Isaacs and Amanda Heslegrave	Approval Date:	**16/02/2022**

	The vial of calibrator stock in PCR tube is for a single use only so discard after use.
7.	Preparation of the samples:<br>a) Take out samples to thaw on ice.<br>b) While samples are thawing, add the required volume of Diluent A to Eppendorf tubes. Place them on ice.<br>c) Once samples thaw, vortex each for 15 sec, spin down briefly, and add the required volume to the already prepared diluent on ice to make up a 1:2 dilution.<br>d) Repeat this for each of the samples.<br>e) Place ready samples on ice.
8.	Loading samples/calibrators on the plate:<br>a) Vortex each sample/calibrator for 15 sec and briefly spin down before loading on the plate.<br>b) To avoid bubbles, use reverse pipetting when loading the samples. Make sure there are no bubbles in the samples because that would hinder the measurement. Duplicates are loaded onto the same well.
9.	Loading reagents in the instrument:<br>a) Go to 'Load Reagent' tab and select a reagent lane.<br>b) Beads: Vortex beads for 30 sec before loading. Scan the barcode of the beads and add available volume in the instrument. Load reagent bottle to one of the three shaking positions in the rack. *These are positions 1-3. Do not let beads sit for idle >5 min. If this happens, vortex again.*<br>c) Detector: Scan the barcode of the detector and add available volume in software. Load reagent bottle in the rack.<br>d) SBG: Scan the barcode of SBG and add available volume in software. Load the reagent bottle in the rack.<br>e) Once all bead, detector, and SBG are loaded in the reagent rack, push the reagent rack in the appropriate lane in the instrument.<br>f) RGP: Select an RGP lane. Scan the barcode of the RGP reagent. Take off the cap and load in the reagent rack. Load the reagent rack in the appropriate lane. *The vial of RGP is for single-day use only. Please discard any remaining at the end of the day.*<br>g) Click 'Done Loading Reagents'.
10.	Creating plate outline and loading plate in the instrument:<br>a) Go to 'Setup Run' tab.<br>b) Assign a batch name (a run name) and assign plate barcode (plate can either be scanned or just be a random number for homebrew assays). Click 'Enter'.<br>c) Assign calibrators: Select 'Assign calibrators' tab. Select a single well and assign the appropriate Homebrew assay. Select the highest calibrator (Calibrator A) and then click 'Descending'. This will assign the rest of the calibrators to the wells below. Set the appropriate number of replicates per well (here: duplicates).<br>d) Assign samples: Select 'Assign samples' tab. Select all the wells that contain samples. Assign the appropriate assay. Set the appropriate number of replicates per well (here: duplicates). Set dilution of samples to 'neat'.<br>e) Place the plate on the plate rack and insert it to the appropriate lane.<br>f) Touch 'Done with set up'.
11.	Check system resources. If required fill System Wash buffer 1, System Wash buffer 2, System liquid or load cuvettes, pipettes tips, or discs. If required, empty solid waste or liquid waste.

12.	If system resources are met, click ‘Start run’ in ‘System resources’ tab. If button is not active, check for further flags in ‘Resource details’ tab.
13.	If the run was started, go to ‘Current run’ tab to monitor assay run.

Table 8: Procedure 3 – After run: Data extraction and post-run maintenance

Step	Step Description
1.	Once the run is finished and the instrument is ‘Ready’, you can proceed with data extraction.
2.	<u>CSV file extraction:</u><br>a) Go to ‘History and Reports’ and click on ‘Run history’ tab.<br>b) Filter results based on ‘Batch ID’.<br>c) Click ‘Select all results’ and then ‘Export’. Select the location where you want to save it, name the file, and press ‘Enter’.<br><u>Batch calibration report extraction:</u><br>a) Go to ‘History and Reports’ and click on ‘Reports’ tab.<br>b) Select ‘Batch calibration report’ and select the appropriate batch (run name).<br>c) Click on ‘Preview’ and once the report is in preview, click on ‘Export’.<br>d) Select the appropriate location where files need to be saved, name the file and press enter to export batch calibration report as a pdf.<br>e) Select ‘Done’ to close the tab.<br>*Further data can be extracted, or values can be recalculated. Refer to Homebrew Assay Development Guide from Quanterix.*
3.	Remove the racks from the instrument and discard reagent bottles, RGP, and plates.
4.	Run ‘End of the day’ maintenance task for post-run maintenance. Load a reagent bottle with System wash buffer 1 and load it into position 3 in a reagent rack.
5.	Go to ‘Maintenance’ tab, select ‘End of the day’ maintenance task, and click ‘Run task’.
6.	Push the reagent rack to lane 4 and click ‘Next’ in ‘Maintenance’ tab. *Wait until the maintenance task is finished, ~15 min.*
7.	Once ‘End of the day’ maintenance task finished, the instrument can be switched off.
8.	Switching off:<br>a) Shutdown software by clicking on 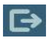 and select ‘Shut down’. This will close the software.<br>b) Shut down the computer then switch off the instrument.
